# PKMYT1 kinase ameliorates cisplatin sensitivity in osteosarcoma

**DOI:** 10.1038/s41392-025-02250-7

**Published:** 2025-05-21

**Authors:** Binfeng Liu, Wei Li, Wenchao Zhang, Chengyao Feng, Lu Wan, Shasha He, Ruiling Xu, Zheng Fu, Zhongyue Liu, Haodong Xu, Xin Jin, Chao Tu, Zhihong Li

**Affiliations:** 1 Department of Orthopedics, The Second Xiangya Hospital, Central South University, Changsha, China; 2 National Clinical Research Center for Mental Disorders, and National Center for Mental Disorders, The Second Xiangya Hospital of Central South University, Changsha, China; 3 Hunan Key Laboratory of Tumor Models and Individualized Medicine, The Second Xiangya Hospital, Changsha, China; 4 Hunan Engineering Research Center of AI Medical Equipment, The Second Xiangya Hospital of Central South University, Changsha, Hunan 410011 China; 5 Department of Urology, The Second Xiangya Hospital, Central South University, Changsha, Hunan China; 6 Department of Oncology, The Second Xiangya Hospital, Central South University, Changsha, Hunan People’s Republic of China; 7 Xinyi Biotech Co., Ltd, Lingang, Shanghai, 201306 PR China; 8 Department of Neurosurgery, The Second Xiangya Hospital of Central South University, Changsha, Hunan China

**Keywords:** Bone cancer, Sarcoma, Cancer therapy

## Abstract

Cisplatin (DDP) remains a cornerstone therapy for osteosarcoma (OS); however, pervasive resistance severely limits its clinical efficacy and worsens patient outcomes. Developing strategies to enhance the chemotherapeutic responsiveness of OS cells is therefore of critical importance. Here, we conducted a kinome-wide clustered regularly interspaced short palindromic repeats (CRISPR) screen, coupled with transcriptome sequencing, to identify regulators of DDP sensitivity. This approach revealed protein kinase membrane-associated tyrosine/threonine 1 (PKMYT1) as a key regulator of DDP sensitivity in OS. Subsequent analysis of patient-derived clinical specimens, along with in vitro functional assays, demonstrated that DDP treatment induces the activation of PKMYT1 in OS cells. Importantly, PKMYT1 silencing markedly enhances cellular sensitivity to DDP, indicating its role in promoting chemoresistance. Mechanistically, PKMYT1 induces phosphorylation of nucleophosmin 1 (NPM1) at the S260 site, which competitively impairs NPM1 SUMOylation. This modification interferes with the recruitment of essential DNA damage response factors, including breast cancer suppressor gene 1 (BRCA1), receptor-associated protein 80 (RAP80), and RADiation sensitive protein 51 (RAD51), ultimately affecting double-strand break (DSB) repair. Furthermore, the selective PKMYT1 inhibitor RP6306 was found to synergize with DDP, amplifying its cytotoxic effects in OS cells. Collectively, these findings highlight PKMYT1 as a promising therapeutic target and provide a rationale for combinatorial strategies to overcome DDP resistance in OS.

## Introduction

Osteosarcoma (OS) is the most common primary malignant bone tumor in children and adolescents. Approximately 15% of OS patients present with metastatic disease at the time of initial diagnosis, with 85% of distant metastatic lesions occurring in the lung tissue, leading to a poor prognosis.^1–3^ Since the 1980s, the treatment of OS has evolved from simple high amputation to comprehensive approaches combining limb-salvaging surgery and neoadjuvant chemotherapy, resulting in an increase in the 5-year survival rate from 17% to 60–70%.^4–6^ Currently, the clinical treatment for OS primarily involves a combination of surgery and neoadjuvant chemotherapy. Cisplatin (DDP), one of the frontline drugs for neoadjuvant and adjuvant chemotherapy in OS, often leads to acquired resistance in most OS patients during chemotherapy, which has become a major obstacle in improving treatment outcomes for OS patients.^7–9^ Therefore, there is an urgent need to identify treatment targets that can increase cisplatin sensitivity in OS, which may help effectively advance the level of OS treatment.

Clustered regularly interspaced short palindromic repeats (CRISPR) screening is a high-throughput technique that involves the design synthesis and cloning of single guide RNAs (sgRNAs) to create a viral vector-based sgRNA library.^10^ This system has been widely utilized for random mutagenesis screening, particularly for identifying mechanisms of drug resistance and for positive or negative selection of candidate genes.^11^ Kinases that are frequently mutated in the genome are closely associated with cancer growth, metastasis, and drug resistance.^12,13^ However, no studies have utilized CRISPR screening to investigate DDP sensitivity in OS. Therefore, employing a CRISPR kinase library to screen for targets that increase DDP sensitivity holds promise in uncovering new therapeutic strategies for OS.

Protein kinase membrane-associated tyrosine/threonine 1 (PKMYT1), a member of the Wee kinase family, regulates the cell cycle by inhibiting Cyclin Dependent Kinase 1 (CDK1). It functions this by phosphorylating CDK1 at T14 and Y15 or by associating with the CDK1/Cyclin B complex to restrict its nuclear localization. This inhibition prevents the G2-to-M phase transition, thereby exerting a negative regulatory effect on cell cycle progression.^14–16^ Notably, recent studies have shown that PKMYT1 plays a crucial role in the occurrence and development of various malignant tumors. For instance, Hu et al. reported that PKMYT1, which functions as a downstream target gene of AlkB homolog 5, RNA demethylase (ALKBH5), undergoes m6A modification, enabling insulin-like growth factor 2 binding protein 3 to recognise and bind to it. This interaction results in enhanced mRNA stability and elevated PKMYT1 expression, ultimately contributing to the significant acceleration of gastric cancer (GC) metastasis.^17^ Tiebang Kang et al. employed a comprehensive CRISPR-Cas9 kinome screen in OS and uncovered the substantial oncogenic function of PKMYT1 in the development of this malignancy.^18^ Simultaneously, Yang Lu et al. revealed that overexpression of PKMYT1 is linked to a poorer prognosis in OS and exacerbates the malignant advancement of MG63 cells by modulating the NF-κB pathway.^19^ In the context of regulating drug sensitivity, Wang et al. performed whole-genome CRISPR/Cas9 screens that identified PKMYT1 as a potential therapeutic target in the oncogenesis and drug resistance of pancreatic ductal adenocarcinoma.^20^ Furthermore, other studies have demonstrated the role of targeting the G2/M checkpoint, particularly through the inhibition of PKMYT1 or WEE1, in sensitizing tumor cells to platinum-based agents.^21–23^ Nevertheless, the role of PKMYT1 in OS drug resistance and its underlying mechanisms remain unclear.

Herein, we employed CRISPR screening to uncover PKMYT1 as a pivotal player in modulating DDP sensitivity in OS. Notably, we observed a substantial upregulation of PKMYT1 in response to DDP treatment in both cell lines and tissue samples. Mechanistically, PKMYT1-induced nucleophosmin 1 (NPM1) S260 phosphorylation competes with NPM1 SUMOylation, affecting the recruitment of DNA repair proteins such as breast cancer suppressor gene 1 (BRCA1), receptor-associated protein 80 (RAP80), and RADiation sensitive protein 51 (RAD51), thereby promoting efficient double-strand break (DSB) repair and ultimately affecting the sensitivity of OS to DDP. Overall, our findings demonstrate for the first time that PKMYT1 overexpression is associated with chemotherapy resistance and poor prognosis in OS, and targeting PKMYT1 significantly enhances the sensitivity of OS cells to DDP, presenting a potential treatment strategy.

## Results

### CRISPR kinase library screening combined with transcriptome sequencing uncovered the pivotal role of PKMYT1 in determining the sensitivity of OS to DDP

**Fig. 1:**
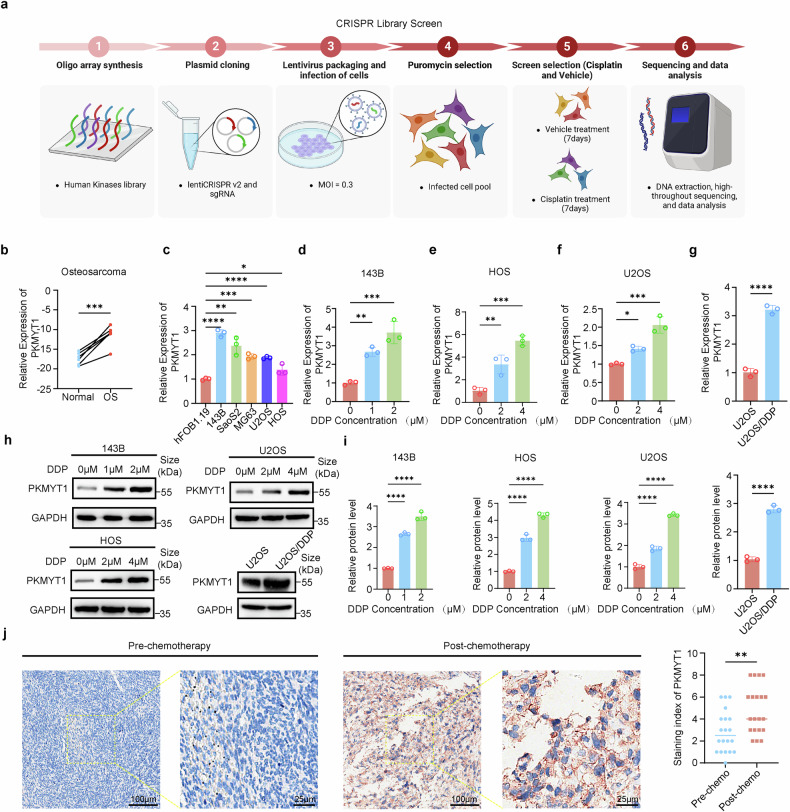
CRISPR kinase library screening combined with transcriptome sequencing identifies PKMYT1 as a key determinant of the Sensitivity of OS to DDP. **a** Schematic representation of the CRISPR kinase library screening protocol. **b** The mRNA expression of PKMYT1 in six paired OS and adjacent normal tissues. P values were assessed by the t test. **c** The expression of PKMYT1 was assessed in osteoblast-differentiated hFOB 1.19 and OS cells (143B, SaoS2, MG63, U2OS, and HOS) using RT-qPCR. The mRNA expression level of PKMYT1 in 143B (**d**), HOS (**e**), and U2OS (**f**) after treatment with different concentrations of DDP for 24 h. **g** The difference in PKMYT1 mRNA expression levels between the DDP-resistant cell line U2OS/DDP and the parental cell line U2OS. **h**–**i** The expression of PKMYT1 in 143B, HOS, and U2OS cells after treatment with varying concentrations of DDP for 24 h, as well as the difference in PKMYT1 protein expression between the DDP-resistant cell line U2OS/DDP and the parental cell line U2OS, was assessed using Western blot analysis. **j** IHC staining of PKMYT1 was performed on 20 osteosarcoma (OS) tissue samples, including both post-chemotherapy and pre-chemotherapy tissues. Representative images are shown, and quantification was analyzed using a t test. Data shown are mean ± SD Error bars, *p < 0.05, **p < 0.01, ***p < 0.001, ****p < 0.0001

To uncover the critical kinases influencing DDP sensitivity in OS, we conducted a CRISPR-Cas9 library screen in 143B cells following the specified procedure (Fig. 1a). We hypothesized that ablation of genes affecting DDP sensitivity would enhance the cytotoxic effects of DDP on tumors. During DDP treatment, cells with sgRNAs targeting key sensitivity-related genes underwent negative selection. Following the screening process, the collected and sequenced cells exhibited a coverage of over 500x, with 100% sgRNA retention in N, N-dimethylformamide (DMF)-treated ground cell, validating the efficacy of screening. Supplementary Fig. 1a shows the top 20 negatively enriched kinases. To further select candidates for subsequent studies, we performed differential expression analysis based on transcriptome sequencing of OS tissues. We identified 503 genes that were significantly upregulated in the OS tissues (Supplementary Fig. 1b). By intersecting the above two datasets, we found that PKMYT1 was the most significantly upregulated in OS tissues and showed substantial negative enrichment in the cells of the DDP treatment group (Supplementary Fig. 1c-f). Moreover, we observed that PKMYT1 was significantly upregulated and associated with poor prognosis in OS based on the Gene Expression Omnibus (GEO) and Therapeutically Applicable Research to Generate Effective Treatments (TARGET) public databases (Supplementary Fig. 1g–i). Although previous studies have revealed that PKMYT1 is closely related to OS proliferation, its relationship with DDP sensitivity remains unknown. Therefore, we further explored the association between PKMYT1 and DDP sensitivity in OS. Initially, we examined the expression of PKMYT1 in six pairs of OS and normal tissues, as well as in OS cell lines (143B, SaoS2, MG63, U2OS, and HOS) using RT-qPCR. The results indicated that PKMYT1 expression was significantly elevated in both OS tissues and cell lines, which was consistent with our previous analysis (Fig. 1b, c). Intriguingly, DDP treatment led to increased PKMYT1 mRNA and protein levels in OS cell lines. Consistently, PKMYT1 was also significantly elevated in the DDP-resistant cell line U2OS/DDP compared with the parental cell line U2OS, implying DDP-induced PKMYT1 activation (Fig. 1d–i). Additionally, IHC results revealed a more significant increase in PKMYT1 expression in OS tissues following chemotherapy than in pre-chemotherapy tissues (Fig. 1j). To investigate the mechanism underlying the increased PKMYT1 levels following DDP treatment, we analyzed the expression of TEAD4, a known transcription factor for PKMYT1 and a promoter of DDP resistance.^24,25^ Our results indicated that TEAD4 expression levels gradually increased after DDP treatment. Furthermore, the knockdown of TEAD4 significantly reduced PKMYT1 expression, while TEAD4 overexpression led to an upregulation of PKMYT1 levels (Supplementary Fig. 2). These results indicate that TEAD4 is essential for regulating PKMYT1 expression during DDP treatment; potentially contributing to the observed increase in PKMYT1 levels and its role in DDP resistance. Collectively, these results suggest a crucial role for PKMYT1 in its impact on the cytotoxic effects of DDP on OS.

### PKMYT1 deficiency increases OS sensitivity to DDP

**Fig. 2:**
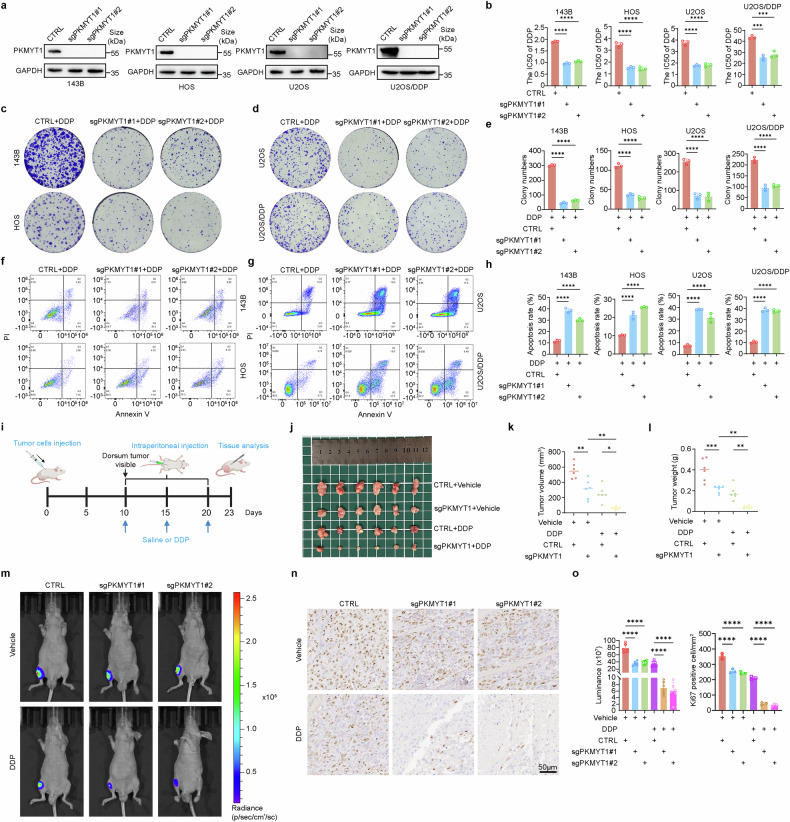
PKMYT1 promotes DDP resistance in OS in vitro and in vivo. **a** The Efficiency of sgRNA Mediated PKMYT1 knockout in 143B, HOS, U2OS, and U2OS/DDP was determined by Western blot. **b** The IC50 of 143B, HOS, U2OS, and U2OS/DDP was calculated after the addition of gradient concentration of DDP for 24 h. P values were assessed by the one-way ANOVA. **c**, **d** The OS cells from A were subjected to treatment with DDP for 7–15 days (143B:0.2 μM, HOS and U2OS:0.4 μM, U2OS/DDP: 1 μM). Cell survival was determined by colony formation assay. **e** Quantification of clone formation number in PKMYT knockout cells treated with DDP. Error bars represent ± SD from three independent experiments. **f**–**g** Apoptosis analysis of PKMYT1 knockout cells treated with DDP for 24 h (143B:1 μM, HOS and U2OS:2 μM, U2OS/DDP: 10 μM). **h** Quantification of apoptosis rate of PKMYT knockout cells treated with DDP. Error bars represent ± SD from three independent experiments. **i** Schematic diagram of tumor xenograft experiments using PKMYT1 knockdown OS cells (143B). 5×10^6^ cells were subcutaneously injected into nude mice. Tumor volumes were measured at indicated days. **j** Representative images of tumors in each group after surgical resection. **k** Tumor volume measured on the indicated day is represented as mean ± SD. **l** Tumor weights were measured and represented as mean tumor weight ± SD. **m**–**o** PKMYT1 knockdown U2OS/DDP-Luc cells were orthotopically injected into mice. Starting on day 10, mice were treated with DDP (3.5 mg/kg) by i.p. injection once every 4 days for a total of three injections. **m** Representative images of living imaging of xenograft mouse model. **n** Representative immunohistochemical images Ki67 in the indicated xenografts tumors from (**m**). **o** Automated quantification of bioluminescence and Ki67 in the indicated xenografts tumors from (**m**). Data shown are mean ± SD Error bars, *p < 0.05, **p < 0.01, ***p < 0.001, ****p < 0.0001

Based on the results of CRISPR library screening and PKMYT1 activation following DDP treatment, we further explored the effect of PKMYT1 on OS sensitivity to DDP. We designed sgRNAs targeting PKMYT1 and generated stable PKMYT1 knockout OS cell lines (143B, HOS, U2OS, and U2OS/DDP) (Fig. 2a). Consistent with previous reports, the results of MTT, colony formation, and flow cytometry analyses revealed that PKMYT1 depletion significantly inhibited the viability of OS cells (Supplementary Fig. 3a–j). Subsequently, we observed that PKMYT1 knockout significantly reduced the IC50 value of DDP in both OS cell lines (143B, HOS, and U2OS) and the chemoresistant OS cell line U2OS/DDP, thereby enhancing the inhibitory effect of DDP on these cells (Fig. 2b–e). Meanwhile, PKMYT1 knockout increased the apoptosis rate induced by DDP (Fig. 2f–h). These results suggest that PKMYT1 knockout significantly increases the sensitivity of OS cells to DDP, which is further validated in the metastatic OS cell line ZOS/M (Supplementary Fig. 3k–n).

Additionally, we validated these results using an in vivo model. PKMYT1 knockout and control 143B cells were inoculated into the right dorsal flank of BALB/c nude mice. When the tumor volume reached 50-100 mm^3^, mice were treated with either DDP or saline (Fig. 2i). In the saline-treated group, PKMYT1 deficiency had a modest inhibitory effect on tumor growth. Moreover, in the DDP-treated group, tumors in the PKMYT1 knockout group were significantly smaller and lighter than those in the control group (Fig. 2j–l). Additionally, PKMYT1 knockout tumors exhibited lower Ki67 expression and a higher proportion of TUNEL-positive cells, particularly under DDP treatment (Supplementary Fig. 4). Convincingly, these findings were further confirmed in a tibial orthotopic xenograft OS model established using the U2OS/DDP cell line (Fig. 2m–o). Collectively, our results indicate that PKMYT1 knockout enhances the sensitivity of OS cells to DDP both in vitro and in vivo.

### PKMYT1 induces the phosphorylation of NPM1 at S260

**Fig. 3:**
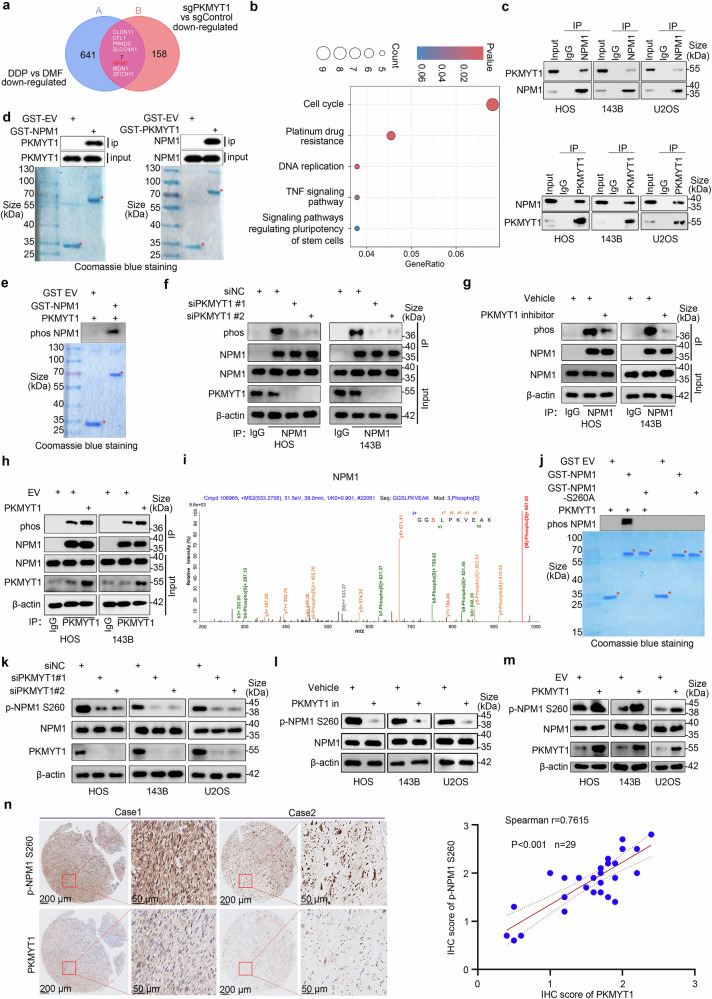
PKMYT1 regulates the phosphorylation of NPM1 at S260. **a** Intersection of phosphorylated molecules downregulated after PKMYT1 knockout and DDP resistance-related genes screened by CRISPR whole genome library. **b** KEGG enrichment analysis results of differentially expressed phosphorylated molecules after PKMYT1 knockout. **c** Co-IP experiments confirm the interaction between PKMYT1 and NPM1, with immunoprecipitation performed using anti-NPM1 and anti-PKMYT1 antibodies on whole-cell lysates, followed by Western blotting with specific antibodies. **d** Western blot (top) analysis of in vitro transcribed and translated PKMYT1 or NPM1 proteins pulled down by GST-NPM1 or GST-PKMYT1 (bottom). **e** GST-pulldown assays evaluating the phosphorylation of NPM1 by PKMYT1. **f** HOS and 143B cells were transfected with siNC, siPKMYT1#1 and siPKMYT1#2 for 48 h. Cells were harvested for co-immunoprecipitation experiments, followed by western blot analysis. **g** HOS and 143B cells were treated with or without RP6306 (2 μM) for 24 h, and cells were harvested for co-immunoprecipitation experiments followed by western blot analysis. **h** HOS and 143B cells were transfected with indicated plasmids for 24 h, and then cells were harvested for co-immunoprecipitation experiments followed by western blot analysis. **i** Phosphorylation peptide map of NPM1 S260. **j** GST-pulldown assays were performed to validate the effect of PKMYT1 on NPM1 S260 phosphorylation. **k**–**m** HOS, 143B and U2OS cells were transfected with indicated siRNAs for 48 h, treated with or without RP6306 (2 μM) for 24 h, and transfected with indicated plasmids for 24 h. and then cells were harvested for co-immunoprecipitation experiments followed by western blot analysis. **n** IHC analysis of the OS tissues (n = 29) by staining the PKMYT1 or NPM1 S260 antibodies. The typical images are shown in panel (**n**). The scale bar indicated in panel n was 200 μm and 50 μm, respectively. The IHC scoring was performed, and the correlation between the IHC score of PKMYT1 and NPM1 S260 was analyzed, with a *p* value as indicated. Data shown are mean ± SD Error bars, *p < 0.05, **p < 0.01, ***p < 0.001, ****p < 0.0001

PKMYT1, a crucial protein kinase, phosphorylates multiple substrate proteins and plays key roles in regulating various biological processes. To elucidate the potential mechanisms by which PKMYT1 influences DDP sensitivity in OS, we aimed to identify PKMYT1-regulated substrate proteins in vivo using phosphoproteomics sequencing. Figure 3b illustrates the pathways enriched by the differentially expressed molecules following PKMYT1 knockout. Additionally, by intersecting the downregulated phosphorylated proteins after PKMYT1 knockout with the results from a CRISPR whole-genome screening for DDP resistance in OS, we identified CLDN11, CFL1, PRKD3, SLCO4A1, NPM1, MDN1, and ZFC3H1 as potential phosphorylation targets through which PKMYT1 may influence DDP sensitivity in OS (Fig. 3a). Among these, NPM1 has been shown to play a significant role in chemotherapy resistance in cancer cells.^26,27^ Therefore, we investigated the association between PKMYT1 and NPM1. Initially, we observed an interaction between PKMYT1 and NPM1 using co-IP and GST pull-down (Fig. 3c and Supplementary Fig. 5). To investigate whether PKMYT1 and NPM1 directly interact, we performed GST pull-down assays using in vitro translated NPM1 or PKMYT1 with GST-PKMYT1 or GST-NPM1, respectively and confirmed a direct interaction between the two proteins (Fig. 3d). Subsequently, GST pull-down assays demonstrated that PKMYT1 could induce the phosphorylation of GST-NPM1 (Fig. 3e). Meanwhile, we observed that silencing PKMYT1 with small interfering RNA (siRNA) or inhibiting its activity using RP6306 significantly reduced NPM1 phosphorylation, whereas overexpressing PKMYT1 led to a marked increase in NPM1 phosphorylation (Fig. 3f–h). Building on our previous phosphoproteomics data, which suggested that PKMYT1 regulates NPM1 phosphorylation at the S260 site, a site essential for NPM1 nucleolar localization,^28^ we generated the NPM1 S260A mutant (phosphodeficient) based on the phosphorylation peptide map of NPM1 S260 and evaluated whether PKMYT1 affects the phosphorylation level at this site (Fig. 3i). Subsequent GST pull-down assays revealed that NPM1 S260A mutant abolished the phosphorylation of NPM1 induced by PKMYT1 (Fig. 3j). Alongside this, we observed that NPM1 S260 phosphorylation levels significantly decreased upon PKMYT1 knockdown or inhibition, whereas overexpression of PKMYT1 notably enhanced NPM1 S260 phosphorylation levels (Fig. 3k–m). Moreover, IHC demonstrated a marked elevation of NPM1-S260 in OS with a significant positive correlation with PKMYT1 (Fig. 3n). Overall, these findings suggest that PKMYT1 binds to NPM1 and promotes phosphorylation at the S260 site of NPM1.

### The phosphorylation of NPM1 at the S260 site is essential for the sensitivity of OS to DDP

**Fig. 4:**
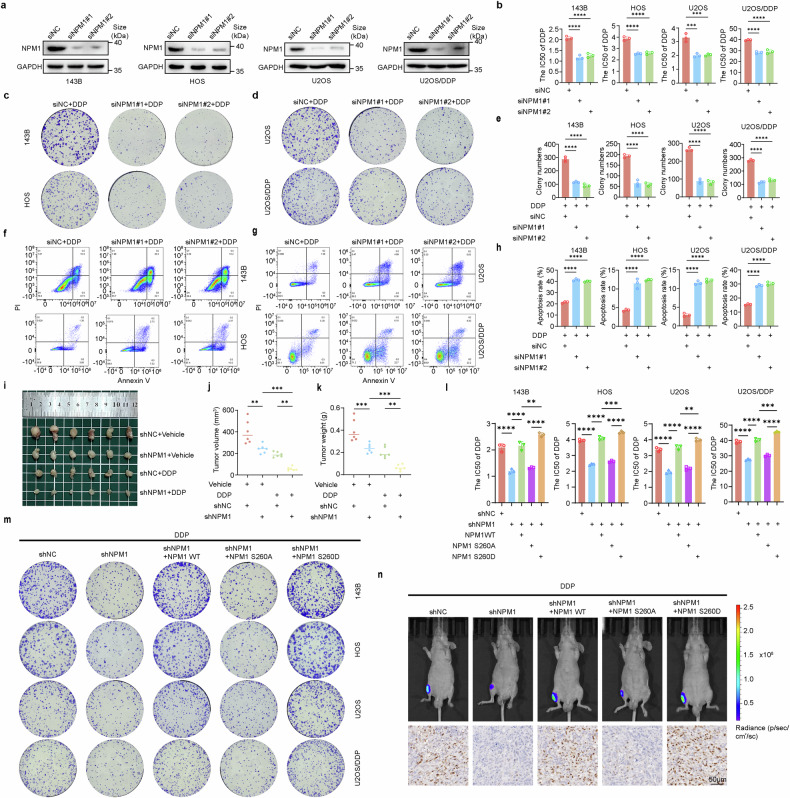
NPM1 mediates DDP sensitivity in OS cells through its phosphorylation at Ser260. **a** The Efficiency of siRNA-mediated NPM1 knockdown in 143B, HOS, U2OS, and U2OS/DDP was determined by WB. **b** The IC50 of OS cell from A was calculated after the addition of gradient concentration of DDP for 24 h. P values were assessed by the one-way ANOVA. **c**, **d** The OS cells from A were subjected to treatment with DDP for 7–15 days (143B:0.2 μM, HOS and U2OS: 0.4 μM, U2OS/DDP: 1 μM). Cell survival was determined by colony formation assay. **e** Quantification of clone formation number in NPM1 knockdown cells treated with DDP. Error bars represent ± SD from three independent experiments. **f**–**g** Apoptosis analysis of NPM1 knockdown cells treated with DDP for 24 h (143B:1 μM, HOS and U2OS:2 μM, U2OS/DDP: 10 μM). **h** Quantification of apoptosis rate of NPM1 knockdown cells treated with DDP. Error bars represent ± SD from three independent experiments. **i**–**k** NPM1 knockdown 143B cells were subcutaneously injected into nude mice. Starting on day 10, mice were treated with DDP (3.5 mg/kg) by i.p. injection once every 4 days for a total of three injections. **i** Representative images of tumors in each group after surgical resection. **j** Tumor volume measured on the indicated day represented as mean ± SD. **k** Tumor weights were measured and represented as mean tumor weight ± SD. **l** The NPM1 knockdown OS cells (143B, HOS, U2OS, and U2OS/DDP) were transiently transfected with the NPM1-WT, NPM1-S260A, and NPM1-S260D. Following the gradient concentration of DDP treatment for 24 h, the IC50 value was determined by MTT assay. **m** The NPM1 knockdown OS cells (143B, HOS, U2OS, and U2OS/DDP) were exogenous expressions of NPM1-WT, NPM1-S260A, and NPM1-S260D, following treatment with DDP for 7–15 days (143B:0.2 μM, HOS and U2OS:0.4 μM, U2OS/DDP: 1 μM). Cell survival was determined by colony formation assay. **n** The NPM1 knockdown OS cells (U2OS/DDP-Luc) were exogenous expressions of NPM1-WT, NPM1-S260A, and NPM1-S260D, following were orthotopically injected into mice. Starting on day 10, mice were treated with DDP (3.5 mg/kg) by i.p. injection once every 4 days for a total of three injections. (Above) Representative images of living imaging of xenograft mouse model. (Below) Representative immunohistochemical images Ki67 in the indicated xenografts tumors. Data shown are mean ± SD Error bars, *p < 0.05, **p < 0.01, ***p < 0.001, ****p < 0.0001

NPM1 is associated with DNA damage repair (DDR) and chemotherapy resistance, but its biological function and clinical significance in OS remain unclear.^26,29^ Consequently, we further explored the effect of NPM1 on the sensitivity of OS to DDP. Initially, we employed siRNA to knock down NPM1 expression in OS cell lines (143B, HOS, U2OS, and U2OS/DDP), as illustrated in Fig. 4a and Supplementary Fig. 6a. We observed that the viability of OS cells was weakened after NPM1 downregulation (Supplementary Fig. 6b–h). The MTT assay results showed that the knockdown of NPM1 significantly reduced the IC50 of DDP, thereby enhancing the sensitivity of OS cells to DDP (Fig. 4b). Additionally, NPM1 interference markedly potentiated the cytotoxic effects of DDP on OS cells (Fig. 4c–e) and increased DDP-induced apoptosis in OS cells (Fig. 4f–h). In vivo, experiments further demonstrated that NPM1 had a certain inhibitory effect on tumor growth. Under DDP treatment, tumors in the NPM1 knockout group showed significantly reduced volume, weight, and Ki-67 positive cells compared to the control group, while the proportion of TUNEL positive cells was higher (Fig. 4i–k and Supplementary Fig. 7). More importantly, to investigate whether the phosphorylation of NPM1 at the S260 site is responsible for OS sensitivity to DDP, we used NPM1 knockdown cells to investigate changes in OS sensitivity to DDP following the exogenous expression of NPM1-WT, NPM1-S260A, and NPM1-S260D (phosphomimetic). The results demonstrated that NPM1 knockdown reduced the IC50 value of DDP in OS cells. Subsequently, exogenous expression of NPM1-WT and NPM1-S260D successfully reversed this effect, whereas expression of NPM1-S260A did not significantly restore the IC50 value (Fig. 4l). These findings were consistently supported by colony formation assays (Fig. 4m and Supplementary Fig. 6i) and further validated in the tibial orthotopic xenograft OS model established with the U2OS/DDP cell line (Fig. 4n and Supplementary Fig. 6j–k). Hence, these findings demonstrated the pivotal role of the phosphorylation of NPM1 at the S260 induced by PKMYT in mediating DDP sensitivity in OS.

### PKMYT1-induced NPM1 S260 phosphorylation promotes efficient DSB repair

**Fig. 5:**
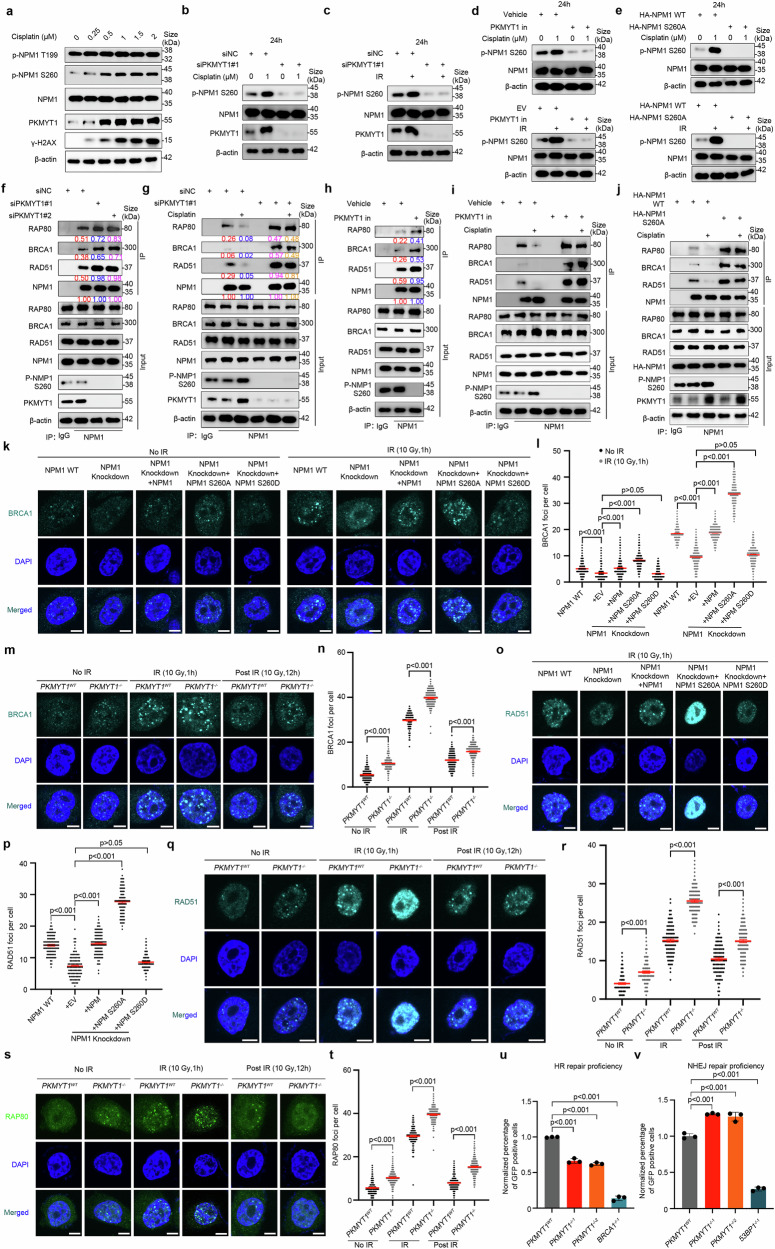
PKMYT1-induced NPM1 S260 phosphorylation promotes efficient DSB repair. **a** Western blot analysis of the expression of PKMYT1, phosphorylated-NPM1 S260, and phosphorylated-NPM1 T199 in OS cells after being treated with various concentrations of DDP. **b**–**d** Western blot analysis of the effect of DDP and IR on NPM1 S260 phosphorylation after PKMYT1 knockdown or inhibition with RP6306. **e** Western blot analysis of the impact of DDP and IR on NPM1 S260 phosphorylation following the overexpression of NPM1 S260 dephosphorylation mutant plasmid. **f** After transfection as indicated siRNAs for 48 h, HEK293T cells were collected and subjected to co-immunoprecipitation experiments followed by western blot analysis. **g** HEK293T cells were transfected with indicated siRNAs for 48 h and treated with or without cisplatin (1 μM) for 24 h before harvesting the cells for co-immunoprecipitation and Western blot. **h** HEK293T cells were treated with or without RP6306 (2 μM) for 24 h before harvesting the cells for co-immunoprecipitation and Western blot. **i** HEK293T cells were treated with or without RP6306 (2 μM) and cisplatin (1 μM) for 24 h before harvesting the cells for co-immunoprecipitation and Western blot. **j** HEK293T cells were transfected with indicated plasmids for 24 h and treated with or without cisplatin (1 μM) before harvesting the cells for co-immunoprecipitation and Western blot. **k**–**l** Immunofluorescence analysis of the effect of NPM1 S260 phosphorylation on IR-induced BRCA1 foci. NPM1 knockout U2OS cells were rescued with either wild-type NPM1, NPM1 S260A mutant and NPM1 S260D mutant, with or without IR (10 Gy) treatment. Representative images are shown in **k**, and the number of BRCA1 foci per group was calculated in l. Approximately 100 cells were counted per group. Data are expressed as the mean ± SEM from three biological replicates. Statistical analysis was performed using Student’s t test, p value as indicated. Scale bar, 10 μm. **m**–**n** Immunofluorescence analysis of PKMYT1 wild-type and PKMYT1 knockout U2OS cells with or without IR (10 Gy) treatment. Representative immunofluorescence images were shown in m, and the number of BRCA1 foci per group was calculated in n, p value as indicated. Scale bar, 10 μm. **o**–**p** NPM1 knockout U2OS cells were rescued with indicated plasmids for 24 h, and immunofluorescence analysis of cells exposed to 10 Gy IR and recovered for 1 h. Representative immunofluorescence images were shown in **o**, and the number of RAD51 foci per group was shown in **p**, p value as indicated. Scale bar, 10 μm. **q**–**r** Immunofluorescence analysis of PKMYT1 wild-type and PKMYT1 knockout U2OS cells with or without IR (10 Gy) treatment. Representative immunofluorescence images were shown in (**q**), and the number of RAD51 foci per group was calculated in **r**. Scale bar, 10 μm. **s**, **t** Immunofluorescence analysis of PKMYT1 wild-type and PKMYT1 knockout cells with or without IR (10 Gy) treatment. Representative immunofluorescence images were shown in (**s**), and the number of RAP80 foci per group was calculated in **t**, p value as indicated. Scale bar, 10 μm. **u** HR assay in PKMYT1 wild-type and PKMYT1 knockout HEK293T cells, with BRCA1 knockout as control. Data are presented as the mean ± SEM from three replicates. Each group included over 1000 cell counts. P value as indicated. **v** NHEJ assay in PKMYT1 wild-type and PKMYT1 knockout HEK293T cells, with 53BP1 knockout as control. Data are presented as the mean ± SEM from three replicates. Each group included over 1000 cell counts, p value as indicated

These results demonstrated that DDP treatment could upregulate PKMYT1 expression, but whether it also increases NPM1 S260 phosphorylation remains unknown. Therefore, we investigated the effect of DDP treatment on NPM1 S260 phosphorylation. We observed that following DDP treatment, the phosphorylation level of NPM1 S260 increased in correlation with the upregulation of PKMYT1 expression, whereas the phosphorylation level at the known NPM1 phosphorylation site T199 remained unchanged (Fig. 5a). Furthermore, PKMYT1 knockdown or inhibition with RP6306 abolished the effect of DDP and ionizing radiation (IR) on NPM1 S260 phosphorylation levels (Fig. 5b–d). Similarly, overexpression of an NPM1 S260A mutant plasmid prevented DDP and IR-induced NPM1 phosphorylation (Fig. 5e). These observations suggest that PKMYT1 upregulation induced by DDP is essential for NPM1 S260 phosphorylation.

DDP exerts anti-cancer effects by inducing DNA damage and inhibiting the proliferation of rapidly dividing cells. However, tumor cells can evade cell death by modulating their DNA damage repair capacity, thereby altering their sensitivity to chemoradiotherapy.^30^ Previous studies have demonstrated that NPM1 SUMOylation regulates homologous recombination (HR) and non-homologous end joining (NHEJ) by recruiting DNA damage repair proteins such as RAD51, RAP80, and BRCA1, thus influencing tumor chemosensitivity.^26^ Specifically, upon DNA DSB, NPM1 SUMOylation facilitates the recruitment of RAD51 to the damage sites, a critical step in homologous recombination, which is essential for DSB repair. Additionally, NPM1 regulates the localization of RAP80, ensuring the proper assembly of the BRCA1 complex at the damage site, thereby affecting the efficiency of DNA repair. Based on these findings, we investigated whether NPM1 S260 phosphorylation is necessary for the regulation of DNA damage protein recruitment. To investigate this, we explored the relationship between NPM1 and DNA repair proteins, including BRCA1. Initially, we observed that neither change in NPM1 expression nor its S260 phosphorylation status affected BRCA1 expression levels (Supplementary Fig. 8a, b). Subsequent Co-IP experiments demonstrated that knocking down PKMYT1 with siRNA or inhibiting it with RP6306 enhanced the interaction of NPM1 with RAD51, RAP80, and BRCA1 (Fig. 5f, h). Conversely, DDP treatment weakened the interaction between NPM1 and these DNA repair proteins, whereas PKMYT1 knockdown or inhibition with RP6306 further abrogated the impact of DDP on these interactions (Fig. 5g, i). Notably, we learn that expression of NPM1 S260A in place of wild-type NPM1 abrogates the ability of DDP to reduce the association between NPM1 and RAP80, BRCA1, and RAD51 (Fig. 5j). These results indicate that phosphorylation of NPM1 at S260 is crucial for regulating its interaction with DNA damage repair proteins.

To better understand the role of NPM1 and its phosphorylation in the recruitment of DNA repair proteins, we generated an NPM1 knockdown cell line and assessed BRCA1 foci formation after IR and DDP treatment. We observed that NPM1 deficiency significantly impaired BRCA1 recruitment to sites of DNA damage, and this defect was rescued by the introduction of wild-type NPM1. Notably, the introduction of the phosphodeficient NPM1 S260A mutant plasmid further enhanced BRCA1 recruitment to damaged sites, whereas the phosphomimetic NPM1 S260D mutant failed to restore BRCA1 recruitment. These effects were more pronounced following IR and DDP treatment (Fig. 5k–l and Supplementary Fig. 8c, d). Subsequently, we evaluated the frequency of BRCA1 foci in PKMYT1-deficient and wild-type cells at various time points following IR treatment. In the resting state, PKMYT1-deficient cells exhibited more BRCA1 foci than wild-type cells did. Following IR and DDP treatment, BRCA1 foci were more abundant in PKMYT1-deficient cells than in wild-type cells. One hour after IR and DDP treatment, PKMYT1-deficient cells showed a marked increase in BRCA1 foci at the damage sites compared to wild-type cells, suggesting that PKMYT1 plays a moderate role in regulating DNA damage foci during the early phase of repair. Interestingly, 12 h after recovery, PKMYT1-deficient cells still displayed a higher number of BRCA1 foci than wild-type cells, indicating that PKMYT1 affects the detachment of BRCA1 from the damaged sites in the later stages of repair (Fig. 5m, n and Supplementary Fig. 8e, f). RAD51 plays a pivotal role in the HR pathway. Following DSBs, RAD51 facilitates the invasion of single-stranded DNA (ssDNA) into undamaged homologous double-stranded DNA (dsDNA).^31^ To further evaluate the role of NPM1 S260 phosphorylation in the end-resection process, we examined its impact on the interaction between NPM1 and RAD51. The results demonstrated that NPM1 deficiency significantly reduced RAD51 recruitment to DNA damage sites after IR and DDP treatment, an effect rescued by wild-type NPM1 or the phosphodeficient NPM1 S260A mutant plasmid but not by the phosphomimetic NPM1 S260D mutant (Fig. 5o, p and Supplementary Fig. 8g, h). Also, we observed that RAD51 recruitment was enhanced in PKMYT1-deficient cells compared to wild-type cells, and even 1 h after IR and DDP treatment, as well as following 12 h of recovery, RAD51 recruitment at lesion sites remained elevated, indicating that PKMYT1 regulates the recruitment and dissociation of RAD51 during damage repair, thereby affecting HR efficiency (Fig. 5q, r and Supplementary Fig. 8i, j). BRCA1 recruitment is typically considered a milestone event in the HR pathway, with RAP80 and CtIP serving as critical factors in the BRCA1-A and BRCA1-C complexes, respectively, and playing distinct roles.^32,33^ The formation of the BRCA1-C complex, comprising CtIP and BRCA1, facilitates end resection and the subsequent HR process. In contrast, the Abraxas/RAP80/BRCA1 complex in BRCA1-A inhibits HR. Consistent with the pattern observed in Fig. 5m, n, PKMYT1 knockout significantly increased the recruitment of RAP80 foci, with this effect being more pronounced after IR and DDP treatment and subsequent recovery. These findings suggest that PKMYT1 regulates the end resection process by selectively influencing RAP80, a key component of the BRCA1 complex, thereby impacting the efficiency of HR (Fig. 5s, t and Supplementary Fig. 8k, l). Furthermore, we aimed to explore whether PKMYT1-induced phosphorylation at the S260 site of NPM1 influences the abundance of DNA repair proteins in a manner related to cell cycle distribution. To investigate this, we performed flow cytometry, which revealed that PKMYT1 ablation inhibited cell cycle progression and increased the proportion of cells in the G2/M phase. This effect may be associated with G2/M checkpoint dysfunction, premature mitotic entry, and the accumulation of genomic instability and DNA damage.^34–36^ Additionally, knockdown of NPM1 led to cell cycle arrest at the G1 phase, whereas the phosphorylation status of the NPM1 S260 site (including NPM1 S260A/D mutants) had no impact on cell cycle progression (Supplementary Fig. 9). These findings suggest that PKMYT1 dynamically regulates the phosphorylation of NPM1 at the S260 site, thereby specifically mediating the recruitment of DNA repair proteins in a manner independent of cell cycle arrest.

Given the role of PKMYT1 in recruiting DNA damage repair proteins and the distinct functions of these proteins in the repair process, we further explored the impact of PKMYT1 on the efficiency of the two major DSB repair pathways: HR and NHEJ. In the HR repair pathway, PKMYT1 depletion caused a notable reduction in the proportion of GFP-positive cells. Conversely, in the NHEJ pathway, PKMYT1 knockout led to a significant increase in the relative percentage of GFP-positive cells (Fig. 5u, v). This suggests that the loss of PKMYT1 impairs HR efficiency, which is subsequently compensated for by augmentation in NHEJ repair. More importantly, we observed that the NPM1 knockout cells exhibit impaired HR and NHEJ repair. Simultaneously, the reduction in HR can be rescued by the exogenous expression of either NPM1-WT or NPM1-S260D, while the decline in NHEJ can be restored by the expression of NPM1-WT or NPM1-S260A (Supplementary Fig. 8m, n). Collectively, these findings highlight the critical role of PKMYT1-induced NPM1 S260 phosphorylation in the effective repair of DSBs, which further modulates DDP sensitivity in OS.

### PKMYT1-induced NPM1 S260 phosphorylation regulates NPM1 deSUMOylation during DSB repair

**Fig. 6:**
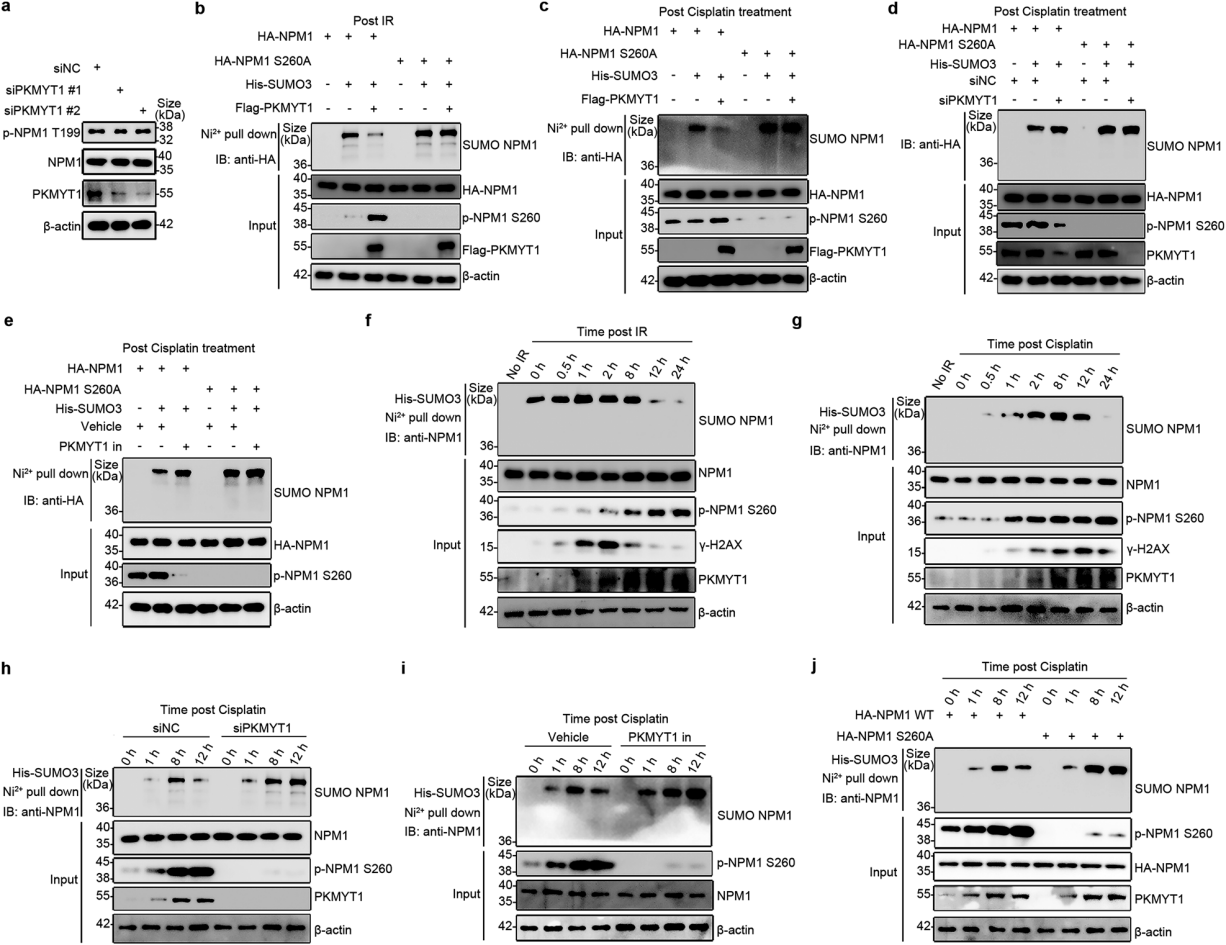
PKMYT1-induced NPM1 S260 phosphorylation regulates NPM1 deSUMOylation during DSB repair. **a** U2OS cells were transfected with indicated siRNAs for 48 h before harvesting the cells for Western blot. **b**, **c** U2OS cells were transfected with indicated plasmids and subjected to His-SUMO pull-down analysis to access the level of NPM1 SUMOylation. **d** U2OS cells were transfected with indicated plasmids and siRNAs and subjected to His-SUMO pull-down analysis. **e** U2OS cells were transfected with indicated plasmids for 24 h and treated with or without RP6306 (2 μM) for 24 h. Cells were collected and subjected to His-SUMO pull-down analysis to assess the level of NPM1 SUMOylation. **f** HEK293T cells were transfected with His-SUMO3 for 48 h and then treated without or with IR (10 Gy). Cells were collected at different time points after IR treatment and subjected to His-SUMO pull-down analysis to access the level of NPM1 SUMOylation. **g** HEK293T cells were transfected with His-SUMO3 for 48 h and then treated without or with cisplatin (2 μM). Cells were collected at different time points after cisplatin treatment and subjected to His-SUMO pull-down analysis. **h** HEK293T cells were transfected with His-SUMO3 and either siNC or siPKMYT1 for 48 h and then treated without or with cisplatin (2 μM). Cells were collected at different time points after cisplatin treatment and subjected to His-SUMO3 pull-down analysis. **i** HEK293T cells were transfected with His-SUMO3 for 48 h and then treated without or with RP6306 (2 μM) for 24 h. Cells were collected at different time points after cisplatin (1 μM) treatment and subjected to His-SUMO3 pull-down analysis. **j** HEK293T cells were co-transfected with His-SUMO3 for 48 h and either NPM1 WT or NPM1 S260A for 24 h, followed by DDP treatment. Cells were collected at various time points to investigate the influence of NPM1 S260 phosphorylation on NPM1 SUMOylation

Previous studies have demonstrated that NPM1 undergoes various posttranslational modifications, including phosphorylation, ubiquitination, and SUMOylation, which are critical for its diverse roles in DNA repair.^37^ Among these, NPM1 SUMOylation is essential for recruiting DNA repair proteins during the DDR, facilitating the assembly of the BRCA1 complex.^26^ This raises the intriguing possibility that NPM1 phosphorylation might influence DSB repair efficiency by interfering with its SUMOylation. We observed that PKMYT1 did not influence the phosphorylation of NPM1 at the T199 site (Fig. 6a). Therefore, our subsequent research focused on the effect of PKMYT1-induced NPM1 S260 phosphorylation on NPM1 SUMOylation. Pull-down assays revealed that PKMYT1 overexpression reduced NPM1 SUMOylation induced by IR and DDP treatment, whereas this effect was abrogated by a dephosphorylation mutant plasmid targeting the NPM1 S260 site (Fig. 6b, c). Additionally, NPM1 SUMOylation increased when PKMYT1 was knocked down via siRNA or inhibited by RP6306, and this increase was reversed by mutating the NPM1 S260 site (Fig. 6d, e). Furthermore, endogenous NPM1 SUMOylation was detectable only during DNA damage and exhibited dynamic changes throughout the DDR process. NPM1 SUMOylation increased with damage, peaking at 1-h post-IR exposure, then declined sharply, becoming nearly undetectable at 12 h (Fig. 6f). Following DDP treatment, NPM1 SUMOylation peaked at 8 h and gradually decreased, becoming nearly undetectable by 24 h (Fig. 6g). In contrast, NPM1 S260 phosphorylation continuously increased during IR and DDP treatments, peaking at 24 h (Fig. 6g). These results suggest a competitive relationship between NPM1 SUMOylation and NPM1 S260 phosphorylation during the DDR. Subsequent experiments showed that NPM1 SUMOylation significantly increased when PKMYT1 was knocked down or inhibited or when NPM1 S260 phosphorylation was blocked using a dephosphorylation mutant plasmid, with SUMOylation peaking at 12 h after DDP treatment (Fig. 6h–j). Collectively, our findings imply that during the DNA damage response, PKMYT1-induced NPM1 S260 phosphorylation competes with NPM1 SUMOylation, affecting the recruitment of DNA repair proteins, such as BRCA1, RAP80, and RAD51, thereby promoting efficient DSB repair and contributing to chemotherapy resistance.

### RP6306 targeting PKMYT1 enhances the sensitivity of OS to DDP

**Fig. 7:**
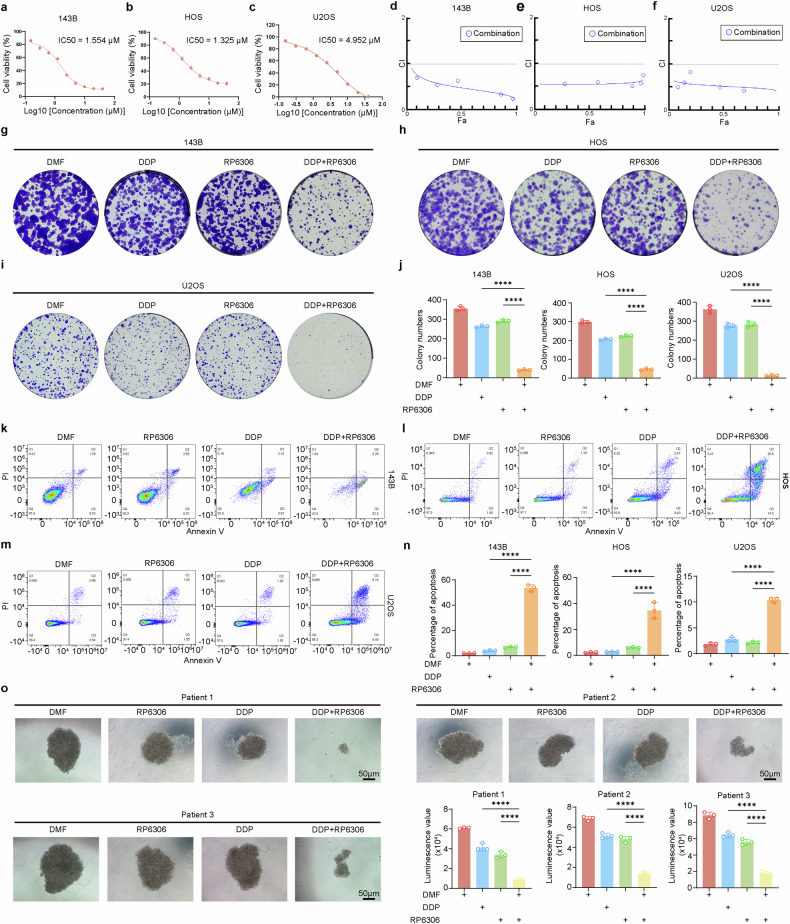
PKMYT1 inhibitor RP6306 synergizes with DDP to inhibit OS. **a**–**c** The IC50 of OS cells (143B, HOS, and U2OS cells) was calculated after the addition of gradient concentration of RP6306 for 24 h. **d**–**f** The Chou-Talalay combination index (CI) was employed to evaluate the synergistic impact of individual drugs or drug combinations on 143B, HOS, and U2OS cell lines. Dose adjustments were made by halving the initial concentrations: DDP at 2 μM was reduced to successive lower concentrations, and RP6306, initially at 1 μM, was similarly diluted. **g**–**i** 143B, HOS and U2OS cells were treated with DPP(0.2 μM), RP6306(0.2 μM), or both for 7–15 days; then, the remaining cells were stained with crystal violet staining. The colony formations were photographed and counted. **j** Quantitative analysis of the clone formation assay results. P values were assessed by the one-way ANOVA. **k**–**m** 143B, HOS, and U2OS cells treated with the indicated concentration of DPP(1 μM), RP6306(1 μM), or both for 24 h and then cell apoptosis rate was determined by Annexin V-FITC/PI assays. **n** Quantitative analysis of the Annexin V-FITC/PI assay results. P values were assessed by the one-way ANOVA. **o** Organoids treated with specified drugs or combinations for 24 h, with viability assessed using the CellTiter-Glo®3D Cell Viability Assay. Data shown are mean ± SD Error bars, *p < 0.05, **p < 0.01, ***p < 0.001, ****p < 0.0001

These results demonstrate that the deficiency of PKMYT1 enhances the efficacy of DDP with a stronger cytotoxic effect, which strongly implies that the molecular targeting of PKMYT1 kinase could be a valuable strategy for inducing chemosensitization to DDP. To validate this hypothesis, we investigated the drug-drug interaction between DDP and the recently discovered PKMYT1 inhibitor, RP-6306.^36^ The Chou-Talalay results indicated that RP6306 and DDP synergistically inhibited the growth of OS at various concentrations, implying that RP6306 can act as a sensitizer for DDP (Fig. 7a–f). Subsequently, the clone formation assay demonstrated that the combination of RP6306 and DDP significantly inhibited OS cell proliferation compared with monotherapy (Fig. 7g–j). Similarly, flow cytometry analysis indicated that the combination of RP6306 and DDP increased the apoptosis rate of OS cells (Fig. 7k–n). Additionally, we treated OS cells with RP6306, DDP, or their combination after NPM1 knockdown and found that NPM1 knockdown abolished the synergistic advantage of combination treatment with DDP alone (Supplementary Fig. 10a). Organoids exhibit heterogeneous characteristics of individual tumors and are widely used as models to simulate clinical responses in vivo. To further validate the efficacy of the combined drug treatment, we established three organoid models based on previous studies, with the clinical treatments of these three patients shown in Supplementary Fig. 10b and Supplementary Table 1. When organoids reached an appropriate size, they were randomly divided into four groups and treated with DMF, RP6306, DDP, or a combination of DDP and RP6306. Interestingly, the combination of DDP and RP6306 significantly inhibited the growth of OS organoids (Fig. 7o). Hence, these data revealed that RP6306 could enhance the sensitivity of OS to DDP, making it a promising adjuvant for improving DDP treatment outcomes.

## Discussion

OS is the most common malignant bone tumor among children and teenagers under 20 years old.^38,39^ The current therapeutic strategy for OS mainly relies on individualized neoadjuvant chemotherapy combined with surgical treatment.^4,40^ However, owing to metastasis and drug resistance, the 5-year survival rate has decreased to 20%.^41,42^ In particular, during chemotherapy, patients may suffer from acquired drug-resistance phenotypes and secondary malignant tumors, which are major obstacles to improving therapeutic efficiency. To address this problem, researchers urgently need to explore the molecular mechanism underlying OS development and how it affects chemotherapy sensitivity to develop new treatment strategies.

PKMYT1 is known to function in cell cycle control. It is a negative regulator of mitosis entry, mainly phosphorylating CDK1 on threonine 14.^43^ The data showed that PKMYT1 plays an essential tumorigenic role in colorectal tumors, hepatocellular carcinoma, and OS and may be an efficient therapeutic target for cancer treatment.^18,44^ However, as a phosphorylating kinase, it remains unknown whether PKMYT1 regulates DDP sensitivity in OS through phosphorylation modification. In our study, we identified PKMYT1 as a vital factor influencing DDP sensitivity in OS through CRISPR screening combined with transcriptome sequencing. Meanwhile, we identified a novel mechanism by which PKMYT1 enhances the efficiency of DSB repair by inducing phosphorylation of NPM1 at the S260 site. This discovery highlights the critical role of PKMYT1 in modulating DDP sensitivity in OS and provides valuable insights into improving chemotherapy outcomes for OS patients.

DDR is a critical cellular process that determines the effectiveness of DDP-induced cancer cell death.^45^ DDP causes extensive DNA damage, including interstrand crosslinks and DSBs. If these lesions remain unrepaired, they lead to replication stress and apoptosis.^46^ The DDR pathway encompasses two main mechanisms for DSB repair: HR, an error-free pathway, and NHEJ, a faster but error-prone process.^47^ The balance between HR and NHEJ plays a pivotal role in regulating cellular sensitivity to chemotherapy.^26^ NPM1 is a highly abundant protein that plays a crucial role in several cellular processes, such as ribosome biogenesis, chromatin remodeling, centrosome duplication, embryogenesis, apoptosis, and DNA repair.^48^ Recent research has increasingly revealed its role in DDR. For instance, SUMOylation of NPM1 affects the recruitment of key DNA repair proteins such as RAD51, BRCA1, and RAP80, leading to higher NHEJ repair efficiency and lower HR repair efficiency, thereby increasing sensitivity to chemotherapy or radiotherapy.^26^ Additionally, NPM1 inhibitors, such as YTR107, disrupt NPM1’s interaction with RAD51, impair HR repair, and sensitize tumors to radiation.^49^ Consistently, our study demonstrated that PKMYT1-induced phosphorylation of NPM1 at S260 competes with its SUMOylation, affecting the recruitment of DNA repair proteins such as BRCA1, RAP80, and RAD51, thereby enhancing efficient DSB repair and mediating DDP sensitivity in OS. These findings imply that targeting NPM1 or its posttranslational modifications could be a promising strategy to overcome chemotherapy resistance in OS. For example, combining DDP with NPM1 inhibitors such as YTR107 may effectively sensitize tumors to treatment, a hypothesis that warrants further investigation.

Combination therapy has shown great potential in overcoming chemotherapy resistance by leveraging the different mechanisms of multiple drugs.^50,51^ This strategy not only enhances anti-cancer efficacy and reduces the risk of resistance but also improves patients’ quality of life. In OS, while platinum-based therapies are commonly used, resistance remains a significant challenge.^52^ Recent studies suggest that CCNE1 amplification may contribute to platinum resistance by disrupting cell cycle regulation. For instance, in bladder and other cancers, CCNE1 amplification has been linked to chemoresistance due to alterations in DNA damage repair pathways, potentially leading to a homologous recombination-proficient phenotype.^22^ Notably, CCNE1 amplification has been associated with synthetic lethality when combined with PKMYT1 inhibition. PKMYT1 negatively regulates CDK1, and its inhibition results in premature mitotic entry and cell death, particularly in CCNE1-amplified cells.^36,53^ Pharmacological inhibitors of PKMYT1, such as RP6306, have been developed and validated in cancers with CCNE1 amplification. RP6306 selectively inhibits PKMYT1 and suppresses tumor growth in CCNE1-amplified cancers.^54^ Known for its oral bioavailability, RP6306 is currently being evaluated in clinical trials, such as the MYTHIC trial (NCT04855656). Early results suggest that RP6306, both as monotherapy and in combination with other treatments, shows promising safety and efficacy, further supporting its potential as a therapeutic option in platinum-resistant cancers. Consistent with these findings, our study demonstrated the synergistic effect of DDP and RP6306 in OS cells and organoid models, highlighting the promise of this combination in overcoming platinum resistance. While these preclinical results are promising, further clinical trials are necessary to evaluate the safety and efficacy of this combination therapy in OS patients.

**Fig. 8:**
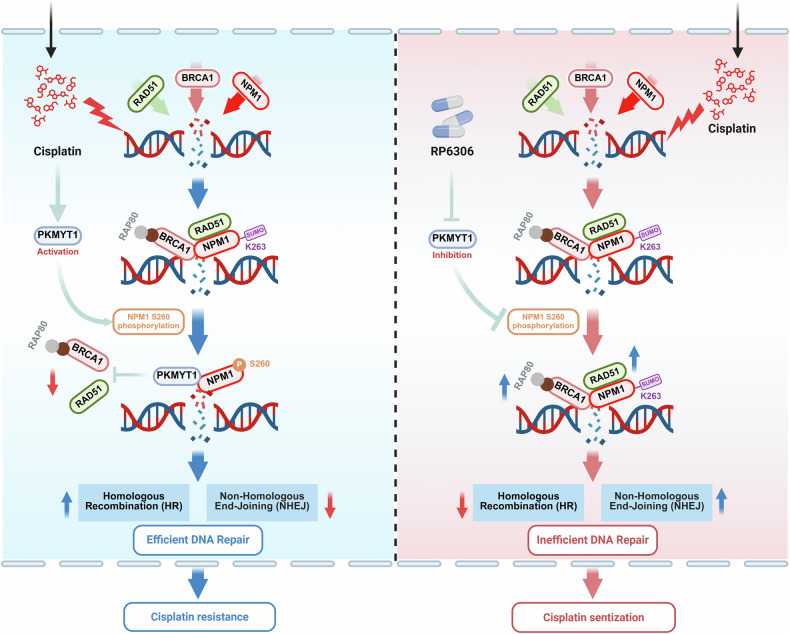
A working model shows that targeting PKMYT1 ameliorates cisplatin sensitivity in OS by regulating the phosphorylation of NPM1. This figure was generated using the online tools of Biorender (https://app.biorender.com/)

In summary, our research indicates that PKMYT1 plays a crucial role in OS sensitivity to DDP. The regulation of DNA damage repair by PKMYT1-induced NPM1 S260 phosphorylation provides new insights into the mechanisms underlying the changes in DDP sensitivity in OS (Fig. 8). Additionally, the combined therapy strategies involving the PKMYT1-targeted inhibitors RP6306 and DDP have significant potential.

## Materials and methods

### Sample collection

This research received approval from the Ethics Committee of the Second Xiangya Hospital, Central South University (approval number: Z0859-01) and complied with the ethical guidelines set forth in the Declaration of Helsinki. Meanwhile, all participants provided written informed consent. As described in our previous study, tumor and adjacent normal tissue samples were obtained from pathologically confirmed OS patients during surgery or biopsy.^51^ Among them, 23 OS samples and 13 paired adjacent normal tissue samples were selected for transcriptome sequencing. The clinical information of the patients and related procedures are as previously described.^51^

### Cell lines and cell culture

The cell lines hFOB1.19, Saos-2, MG63, U2OS, HOS, and HEK293T were purchased from Procell Life Science & Technology Co., Ltd., China. The 143B cell line was purchased from the American Type Culture Collection (ATCC). The DDP-resistant OS cell lines (U2OS/DDP) were developed from the parental U2OS cell line by exposing the parental line in vitro to stepwise the increased DDP concentrations. The ZOS/M line was gifted by Prof. Kang Tiebang (Sun Yat-Sen University, China) and cultured as described previously.^55^ The hFOB1.19 cell line was cultured in DMEM/F12 medium (Procell Life Science & Technology, PM150312) containing 0.3 mg/mL G418 (Procell Life Science & Technology, PB180125) and 10% FBS (NEST Biotechnology). The 143B and HEK293T cell lines were cultured in DMEM (Procell Life Science & Technology, PM150210) supplemented with 10% FBS. The HOS and MG63 cell lines were cultured in MEM (Procell Life Science & Technology, PM150410) containing 10% FBS. The U2OS cell line was cultured in McCoy’s 5 A medium (Procell Life Science & Technology, PM150710) with 10% FBS, whereas the Saos-2 cell line was cultured in McCoy’s 5 A medium containing 15% FBS. All media were supplemented with a 1% Penicillin-Streptomycin Solution (Procell Life Science & Technology, PB180120). All cell lines were incubated at 37 °C with 5% CO₂ in a humidified environment, except for hFOB1.19, which was cultured at 34 °C under the same CO₂ and humidity conditions. The cell lines used in this study were authenticated by short tandem repeat (STR) profiling, which confirmed the absence of no misidentification or cross-contamination with other cell lines. Additionally, the cell lines were tested and were found to be free of mycoplasma contamination.

### Quantitative real-time PCR (RT-qPCR)

Total RNA was extracted from tissues with TRIzol (Cat#9109; Takara Bio) and from cells using an RNA extraction kit (AG21023, ACCURATE BIOTECHNOLOGY, Hunan, China), following the respective manufacturer’s instructions. The RNA concentration and quality were evaluated using a NanoDrop 2000 spectrophotometer (Thermo Fisher Scientific). Next, cDNA was synthesized via reverse transcription using the HiScript IV RT SuperMix for qPCR (Vazyme Biotech Co.,Ltd, R423). Real-time quantitative PCR was subsequently conducted on a QuantStudio 5 system (Applied Biosystems, USA) utilizing the Hieff® qPCR SYBR Green Master Mix (Yeasen Biotechnology Co., Ltd., 11202ES08). GAPDH was used as the internal control, as it is a commonly employed housekeeping gene with stable expression across different cell types and conditions. The relative gene expression levels were determined using the 2^-ΔΔCT^ method, which normalizes the expression of target genes to GAPDH and calculates the fold change relative to the control group.^56,57^ All the specific primer sequences are provided in Supplementary Table 2.

### Co-immunoprecipitation (Co-IP)

After washing the cells three times with PBS, they were lysed using a buffer containing PMSF and a protease inhibitor cocktail (Sigma, St. Louis, MO, USA). Lysis buffer contained 50 mM Tris-HCl (pH 7.4), 150 mM NaCl, 50 mM NaF, 1 mM EDTA, 1 mM Na_2_P_2_O_4_, and 1 mM Na_3_VO_4_. For co-IP of flag-tagged proteins, the lysates were incubated overnight at 4 °C with anti-flag or anti-mouse IgG agarose beads (Sigma-Aldrich). To immunoprecipitate endogenous proteins, whole-cell lysates were incubated with specific antibodies and Protein A/G agarose beads (Thermo Fisher Scientific, Inc., Waltham, MA, USA) under the same conditions. After incubation, the beads were thoroughly washed six times with wash buffer, resuspended in loading buffer, and prepared for western blot analysis.

### Phosphorylation proteomics

Phosphorylation proteomics was performed by Shanghai Bioprofile Biotechnology Co., Ltd. Briefly, 143B cells from the sgCTRL and sgPKMYT1 groups were lysed in 200 μL of lysis buffer (4% SDS, 100 mM DTT, 150 mM Tris-HCl, pH 8.0), boiled, and sonicated. After centrifugation, the supernatant was collected, and protein concentration was determined using a BCA assay. The proteins were processed via FASP, reduced, alkylated, and digested with trypsin, followed by peptide desalting using C18 stage tips. Phosphopeptides were enriched using the High-Select™ TiO_2_ Phosphopeptide Enrichment Kit, applied to TiO_2_ spin tips, washed, and eluted with elution buffer. The eluted phosphopeptides were dried and resuspended in 0.1% formic acid for LC-MS/MS analysis. Peptide separation was carried out on a Vanquish Neo UHPLC system and analyzed on an Orbitrap Astral mass spectrometer using the Data-Independent Acquisition (DIA) method. DIA data were processed with Spectronaut 18 for protein and phosphorylation site identification, and label-free intensity-based quantification was applied. Bioinformatics analysis was performed using Perseus software, R, and KEGG pathway enrichment.

### Glutathione S-transferase (GST) pull-down

Cell lysis was performed using RIPA buffer supplemented with protease and phosphatase inhibitors. The GST-tagged protein (GST-NPM1 and GST-PKMYT1) was captured using glutathione-sepharose beads (GE Health-care Lifesciences). Following incubation with cell lysates at 4 °C for 24 h, the beads were extensively washed with lysis buffer. To confirm the direct interaction between NPM1 and PKMYT1, beads were incubated overnight at 4 °C with in vitro transcribed and translated NPM1 or PKMYT1 (Promega). After incubation, the beads underwent six washes with binding buffer before being resuspended in the sample buffer. The associated proteins were then analyzed via western blotting and Coomassie blue staining.

### Western blot (WB)

Cellular proteins were extracted using radioimmunoprecipitation assay (RIPA) buffer, and their concentrations were quantified using the Micro BCA Protein Assay Kit (New Cell & Molecular Biotech, WB6501). The extracted proteins were subjected to SDS-PAGE and transferred to (lyvinylidene fluorine a PVDF) membranes. The membrane was blocked with nonfat milk at room temperature for 1 h and then incubated overnight at 4 °C with the appropriate primary antibody. The membrane was washed thrice with TBST and incubated with the secondary antibody at room temperature for 1 h. Finally, the PVDF membrane was exposed to a chemiluminescent substrate, and the signal was captured using the Bio-Rad Image Lab system.

The antibodies used in this study are as follows: GAPDH (#60004-1-Ig, Proteintech, 1:10,000 dilution), PKMYT1 (#4282S, Cell Signaling Technology, 1:1,000 dilution), NPM1 (#60096-1-Ig, Proteintech, 1:5,000 dilution), p-NPM1 T199 (#AF3111, Affnity, 1:1000 dilution), p-NPM1 S260 (#TP50488, HUABIO, 1:3000 dilution), p-Histone H2A.X S139 (#P40705, ProMab Biotechnologies Inc., China, 1:1000 dilution), RAP80 (#13642-1-AP, Proteintech, 1:1000 dilution), BRCA1 (#22362-1-AP, Proteintech, 1:1000 dilution), RAD51(#14961-1-AP, Proteintech, China, 1:2000 dilution), anti-HA (#AB0004, Abways, China, 1:2000 dilution), β-actin (A00730, Genscript Biotech, 1:1000 dilution), Phosphoserine/threonine/tyrosine (#11995 R, Yajikit, 1:100 dilution), HRP conjugated goat-anti-mouse antibody (#SA00001-1, Proteintech, 1:10,000 dilution) and HRP conjugated goat-anti-rabbit antibody (#511203, zen-bioscience, 1:5000 dilution).

### Colony formation assay

OS cells (143B, HOS, U2OS, U2OS/DDP, and ZOS/M) were plated in 6-well plates at a density of 1000 cells per well. Following overnight incubation, drug treatments were administered, and the cells were cultured until colonies became visible (7–14 days). After discarding the medium, cells were washed three times with PBS, fixed with 4% paraformaldehyde for 30 min, and stained with crystal violet for the same duration. The staining reagent was then gently rinsed off with PBS, and the plates were air-dried before imaging. Colony quantification was conducted using ImageJ software.

### MTT assay

In short, 1000 suspended cells were seeded into each well of a 96-well plate. Relevant drug treatments were administered, and the assay was performed after incubation at 37 °C with 5% CO2 for the appropriate duration. To perform the assay, 10 μL of MTT reagent (5 mg/mL) (Sigma-Aldrich, M2128) was added to each well, and after 4 h of incubation, the reaction was terminated by adding 100 μL of dimethyl sulfoxide (DMSO) to each well. After 10 min of low-speed shaking, absorbance was recorded at 570 nm using a SpectraMax M5 microplate reader (USA). Cell viability and half maximal inhibitory concentrations (IC50) were calculated based on the measurement results using GraphPad Prism 5.0.

### Transfection of siRNA

The small interfering RNA (siRNA) utilized in this study was procured from Tsingke Biotechnology Co. Specific siRNA sequences are listed in Supplementary Table 3. The experiment was conducted according to the manufacturer’s instructions. Briefly, OS cells were seeded into 6-well plates, and upon reaching a cell density of 30-50%, siRNA was transfected into the cells using Lipofectamine 2000 (Thermo Fisher Scientific, 12566014). Post-transfection, transfection efficiency was assessed via RT-qPCR and Western blotting, and the efficacious siRNA was chosen for subsequent experiments.

### Lentivirus package and stable knockout cell generation

To establish stable PKMYT1 knockout (KO) cell lines, we constructed KO-stable cell lines based on sgRNA. The sgRNAs of PKMYT1 were a gift from Prof. Kang Tiebang (Sun Yat-Sen University, China) and the sequences of the relevant sgRNAs are listed in Supplementary Table 4. shRNA was used to construct stable cell lines with NPM1 knockdown, and the sequences of shNPM1 were identical to those of their siRNA. Lentivirus packaging and transfection were performed according to previously reported methods. Briefly, sgCTRL, sgPKMYT1, and shNPM1 were co-transfected with psPAX2 and PMD2.G into HEK293T cells. Lentivirus particles were collected at 48- and 72-h post-transfection and concentrated using Amicon® ultrafiltration filters (Millipore, USA). Next, the target cells were infected with lentiviral particles overnight in the presence of 10 μg/mL of polybrene. After 24 h of infection, the medium was replaced with fresh medium, and cells were cultured for an additional 24 h. Selection was performed using a fresh medium containing 2 μg/mL puromycin to obtain stable cell lines.

### Pharmacological inhibitors, reagents and plasmids

Cisplatin (HY-17394), the selective PKMYT1 inhibitor RP6306 (HY-145817A), and N, N-dimethylformamide (DMF; HY-Y0345) were purchased from MedChemExpress. A Protease Inhibitor Cocktail (Catalog No. C0001) was obtained from TOPSCIENCE. Synthetic sgRNA sequences were annealed and ligated into the lenti CRISPR V2 vector. The PLKO.1-puro vector was used to clone shRNA targeting NPM1.

### CRISPR/Cas9 library screen

The Kinome CRISPR knockout library employed in this study was custom-designed by SYNBIO Technologies, targeting 507 human kinases with 10 distinct sgRNAs per gene. In addition, 164 non-targeting sgRNAs were included as controls. The CRISPR library screening workflow is shown in Fig. 1a. Briefly, cells were transduced with the Kinome CRISPR library at a low multiplicity of infection (MOI) of 0.3, ensuring 500-fold coverage. Following transduction, cells were selected with 2 µg/mL puromycin for seven days to remove non-transduced cells. The generated mutant cell pool was treated with vehicle (DMF) or 0.25 µM DDP for 10 days. Genomic DNA was then extracted using the DNA Midi Kit (Qiagen), and sgRNAs were amplified with NEBNext® High-Fidelity 2X PCR Master Mix before sequencing by Genegy Biotechnology (Shanghai, China). Data analysis was performed using the MAGeCK v0.5.7 algorithm, following established protocols.^58^

### DNA repair assays

DNA repair assays were performed following established protocols.^26^ For NHEJ assays, the cells were transfected with a linearized pcDNA3.1/puromycin plasmid (Invitrogen) along with the pEGFP-C1 plasmid. After 36 h, cells were harvested, counted, and seeded onto two separate plates. Transfection efficiency was assessed by normalizing the EGFP expression levels. In the HR assays, the cells were co-transfected with the DR-GFP plasmid, I-SceI expression vector, and DsRed plasmid. After 48 h, the cells were collected, washed with 1× PBS, and analyzed by FACS using a FACSVerse instrument (BD Biosciences, USA) to measure green (EGFP) and red (DsRed) fluorescence. The repair efficiency was determined by calculating the ratio of EGFP and DsRed double-positive cells to DsRed-positive cells, normalized to the repair efficiency of wild-type cells. FlowJo software was used to analyze the proportion of GFP-positive cells relative to that of DsRed-expressing cells. U2OS-DR-GFP cells transfected with only DsRed, without I-SceI, served as a negative control to establish the background level of HR. Repair frequencies were normalized to those observed in wild-type cells.

### Immunohistochemistry (IHC)

All clinical specimens of OS used in this study were histopathologically and clinically diagnosed. The use of these specimens adhered to ethical standards. IHC staining was conducted according to the instructions provided with the High-Efficiency IHC Detection System Kit (Absin, abs957). Briefly, after dewaxing, antigen retrieval, inactivation, and blocking, sections were incubated overnight at 4 °C with the following antibodies: PKMYT1 (Proteintech, 67806-1-Ig, 1:500) and Ki67 (Abcam, ab15580, 1:200). Subsequently, the corresponding secondary antibodies were applied, and DAB color development was initiated. The expression of relevant genes in the tissue was scored by two independent observers based on the positive staining ratio and staining intensity. The scoring methods used were consistent with those described in previous research reports.^59^

### Flow cytometry

According to the manufacturer’s instructions, the Annexin V-PE/PI Apoptosis Detection Kit (#40302ES60, YEASEN) was used to measure the rate of cell apoptosis. Briefly, the cells were collected and washed once with pre-chilled PBS. Subsequently, the cells were stained with buffer containing Annexin V and PI for 10 min. Following staining, the reaction was terminated, and the samples were analyzed using a flow cytometer. Finally, data analysis was performed using FlowJo software (FlowJo, USA), and the apoptosis rate was calculated as the sum of the early and late apoptosis rates.

### Animal experiment

All animal experiments in this study were approved by the Institutional Animal Care and Use Committee of the Second Xiangya Hospital, Central South University (approval number: 20230943), and conducted in accordance with established guidelines. Male BALB/c nude mice (4–5 weeks old) were sourced from the Experimental Animal Center of Central South University and maintained in a specific pathogen-free (SPF) barrier facility. To establish a subcutaneous xenograft model, 5 × 10^6^ 143B cells were subcutaneously injected into the right dorsal flank of nude mice. For the orthotopic xenograft model, 1 × 10^6^ U2OS/DDP stable cells expressing Luciferase were implanted into the proximal tibia through the cortex of the anterior tuberosity. Tumor growth was monitored using noninvasive bioluminescence imaging with the IVIS Lumina II platform (PerkinElmer). Ten days after tumor cell inoculation, mice were randomly assigned to receive either DDP (DDP, 3.5 mg/kg, administered via intraperitoneal injection) or saline as a control, administered intraperitoneally once every 4 days for a total of three injections. Following the final injection, the mice were euthanized, and the tumors were excised, weighed, and fixed in 4% paraformaldehyde.

### OS organoid culture

As previously described, we constructed and cultured OS organoids.^60^ All procedures involving human specimens were approved by the Ethics Committee of the Second Xiangya Hospital, Central South University (approval number: Z0859-01). Written informed consent was obtained from all patients. Briefly, fresh OS specimens were washed three times with PBS, cut into small pieces, and further digested. The suspension was filtered to remove the undigested tissue, and the cells were centrifuged to form spheres. These cell clusters were further cultured using a 3D vibrational screening system. Cell viability within the organoids was assessed using the CellTiter - Glo® 3D Cell Viability Assay Kit (Promega, G9681).

### Drug-drug interaction assay

Cell viability of OS cells (143B, HOS, and U2OS) treated with DDP, RP6306, or their combination was assessed using the MTT assay. Then, the synergy between DDP and RP6306 was evaluated using the Chou-Talalay CI method. The CI was calculated using the formula: CI = DA/da + DB/db, where DA and DB are the IC50 values of the individual drugs, and da and db represent the concentrations of each drug required to achieve 50% inhibition when used in combination. The following criteria were used to evaluate drug synergy: CI > 1 indicates antagonism, CI < 1 indicates synergy/cooperation, CI between 0.3–0.7 indicates synergism, and CI between 0.1–0.3 indicates strong synergism.

### Immunofluorescence

Cell slides of the appropriate size were placed in 6-well plates, and OS cell lines were seeded onto the slides. After overnight culture, cells were fixed with 4% paraformaldehyde at room temperature for 30 min, followed by permeabilization with 0.3% Triton X-100 for 30 min. After washing thrice with PBS, the cells were blocked with 5% bovine serum albumin for 1 h at room temperature. The cells were then incubated with the corresponding primary antibodies at 4 °C overnight and subsequently with fluorescent dye-conjugated secondary antibodies at room temperature for 1 h. The cell nuclei were stained with 4,6-diamidino-2-phenylindole (DAPI). Finally, the cell slides were mounted on glass slides, and images were obtained using a fluorescence microscope.

### Tunel assay

Tunel staining was performed using the TUNEL Apoptosis Assay Kit (C1088, Beyotime) according to the manufacturer’s instructions. Nuclei were co-stained with DAPI for 5 min, followed by washing and imaging using a fluorescence microscope. TUNEL-positive cells exhibited green fluorescence.

### Statistical analysis

All data were analyzed using GraphPad Prism 9. Sample sizes are indicated in the Figure legends. The analyzed samples met the appropriate experimental conditions. Statistical significance was assessed using an unpaired Student’s t test for comparisons between two experimental groups and one-way or two-way ANOVA for comparisons involving more than two experimental groups, as appropriate. As described in our previous studies, survival analysis was conducted based on the GSE16091 and GSE21257 cohorts from the GEO database, as well as the TARGET cohorts. The differences in survival times were compared using the log-rank test. DESeq2 was used to analyze RNA-Seq data for differential expression, and enrichment analysis was performed using the KEGG pathway database. Statistical significance was set at P < 0.05. The symbols are as follows: “*“ indicates p < 0.05, “**“ indicates p < 0.01, “***“ indicates p < 0.001, and “****“ indicates p < 0.0001.

## Supplementary information

Supplementary Materials

Original western blots

## Data Availability

The Long-read transcriptome and NGS data have been deposited in GEO, and the accession number was GSE218035. All other data are available from the corresponding author upon reasonable request.
