# Supplementary material for: PKMYT1 kinase ameliorates cisplatin sensitivity in osteosarcoma
Source: Signal Transduct Target Ther. 2025 May 21;10:165. doi: 10.1038/s41392-025-02250-7 (PMC12092789; doi:10.1038/s41392-025-02250-7)
Supplement: Supplementary file 1 — Supplementary Materials [file 41392_2025_2250_MOESM1_ESM.docx]

Supplementary Materials for

PKMYT1 kinase ameliorates cisplatin sensitivity in osteosarcoma

Binfeng Liu^1,2,3,4#^, Wei Li^5,2#^, Wenchao Zhang^1,2,3^, Chengyao Feng^1,2,3^, Lu Wan^1,2,3^, Shasha He^6^, Ruiling Xu^1,2,3^, Zheng Fu^7^, Zhongyue Liu^2,3,8^, Haodong Xu^1,2,3^, Xin Jin^5,2*^, Chao Tu^1,2,3,4*^, Zhihong Li^1,2,3,4*^

^1^ Department of Orthopedics, The Second Xiangya Hospital, Central South University, Changsha, China. ^2^ National Clinical Research Center for Mental Disorders, and National Center for Mental Disorders, The Second Xiangya Hospital of Central South University, Changsha, China. ^3^ Hunan Key Laboratory of Tumor Models and Individualized Medicine, The Second Xiangya Hospital, Changsha, China. ^4^ Hunan Engineering Research Center of Artificial Intelligence Based Medical Equipment, The Second Xiangya Hospital of Central South University, Changsha, Hunan 410011, China. ^5^ Department of Urology, The Second Xiangya Hospital, Central South University, Changsha, Hunan, China. ^6^ Department of Oncology, The Second Xiangya Hospital, Central South University, Changsha, Hunan, People's Republic of China. ^7^ Xinyi Biotech Co., Ltd, Lingang, Shanghai, 201306, PR China. ^8^ Department of Neurosurgery, The Second Xiangya Hospital of Central South University, Changsha, Hunan, China.

Correspondence to: Xin Jin (jinxinxy2@csu.edu.cn) or Chao Tu (tuchao@csu.edu.cn) or Zhihong Li (lizhihong@csu.edu.cn)

**This PDF file includes:**

Table S1 to S4

Fig. S1 to S10

TABLE OF CONTENTS

Supplementary Table 1. The clinical information of OS patients used for constructing organoids1

Supplementary Table 2. The primer sequences used for RT-qPCR1

Supplementary Table 3. The sequences of siRNA1

Supplementary Table 4. The primer sequences for sgRNA1

Supplementary Fig. 1 The relationship between PKMYT1 and OS2-3

Supplementary Fig. 2 DDP regulates PKMYT1 expression by upregulating TEAD44

Supplementary Fig. 3 The impact of PKMYT1 on the proliferative capacity of OS and the validation of its effect on OS to DDP sensitivity in the ZOS/M cell line5-6

Supplementary Fig. 4 PKMYT1 knockdown improves sensitivity of 143B xenografts to DDP7

Supplementary Fig. 5 GST-pulldown assay demonstrating the Interaction between NPM1 and PKMYT18

Supplementary Fig. 6 The impact of NPM1 on the proliferative capacity of OS9-10

Supplementary Fig. 7 NPM1 knockdown affects the sensitivity of 143B xenografts to DDP11

Supplementary Fig. 8 PKMYT1-induced NPM1 S260 phosphorylation promotes efficient DSB repair12-13

Supplementary Fig. 9 The effect of PKMYT1 knockout, NPM1 knockdown, and NPM1 S260 phosphorylation statue on the cell cycle of OS cells. 14

Supplementary Fig. 10 Effect of NPM1 knockdown on the combined treatment of RP6306 and DDP and the clinical information of OS patients used for constructing organoids15

**Supplementary table 1. The clinical information of OS patients is used for constructing organoids.**

| Patient ID | Age at diagnosis | Gender | Histological type | Primary tumor site | Chemotherapy | Metastasis | Chemotherapy Regimen |
| --- | --- | --- | --- | --- | --- | --- | --- |
| 1 | 18 | Female | Conventional osteosarcoma | Lower-extremity | Neoadjuvant chemotherapy | No Metastasis | Doxorubicin, cisplatin, and high-dose methotrexate (MAP) |
| 2 | 39 | Male | Conventional osteosarcoma | Lower-extremity | Neoadjuvant chemotherapy | No Metastasis | Doxorubicin, cisplatin, and high-dose methotrexate (MAP) |
| 3 | 11 | Female | Conventional osteosarcoma | Lower-extremity | Neoadjuvant chemotherapy | No Metastasis | Doxorubicin, cisplatin, and high-dose methotrexate (MAP) |

**Supplementary table 2. The primer sequences used for RT-qPCR.**

| **Gene** | **Primer sequence (5′-3′)** |
| --- | --- |
| GAPDH(F) | CAAGGTCATCCATGACAACTTTG |
| GAPDH(R) | GTCCACCACCCTGTTGCTGTAG |
| PKMYT1(F) | CATGGCTCCTACGGAGAGGT |
| PKMYT1(R) | ACATGGAACGCTTTACCGCAT |
| NPM1(F) | GGAGGTGGTAGCAAGGTTCC |
| NPM1(R) | TTCACTGGCGCTTTTTCTTCA |

**Supplementary table 3. The sequences of siRNA.**

| **Gene** | **Primer sequence (5′-3′)** |
| --- | --- |
| NPM1-NC (sense) | UUCUCCGAACGUGUCACGUTT |
| NPM1-siRNA#1 | GGAAUGUUAUGAUAGGACATT |
| NPM1-siRNA#2 | AGGTGGTAGCAAGGTTCCA |

**Supplementary table 4. The primer sequences for sgRNA.**

| **Gene** | **Primer sequence (5′-3′)** |
| --- | --- |
| sgCTRL-F | CAAGGTCATCCATGACAACTTTG |
| sgCTRL-R | GTCCACCACCCTGTTGCTGTAG |
| sgPKMYT1#1-F | CACCGGGGCCATGGCTCCTACGGAG |
| sgPKMYT1#1-R | AAACCTCCGTAGGAGCCATGGCCCC |
| sgPKMYT1#2-F | CACCGAACATGGAGCTGCCCCACGG |
| sgPKMYT1#2-R | AAACCCGTGGGGCAGCTCCATGTTC |


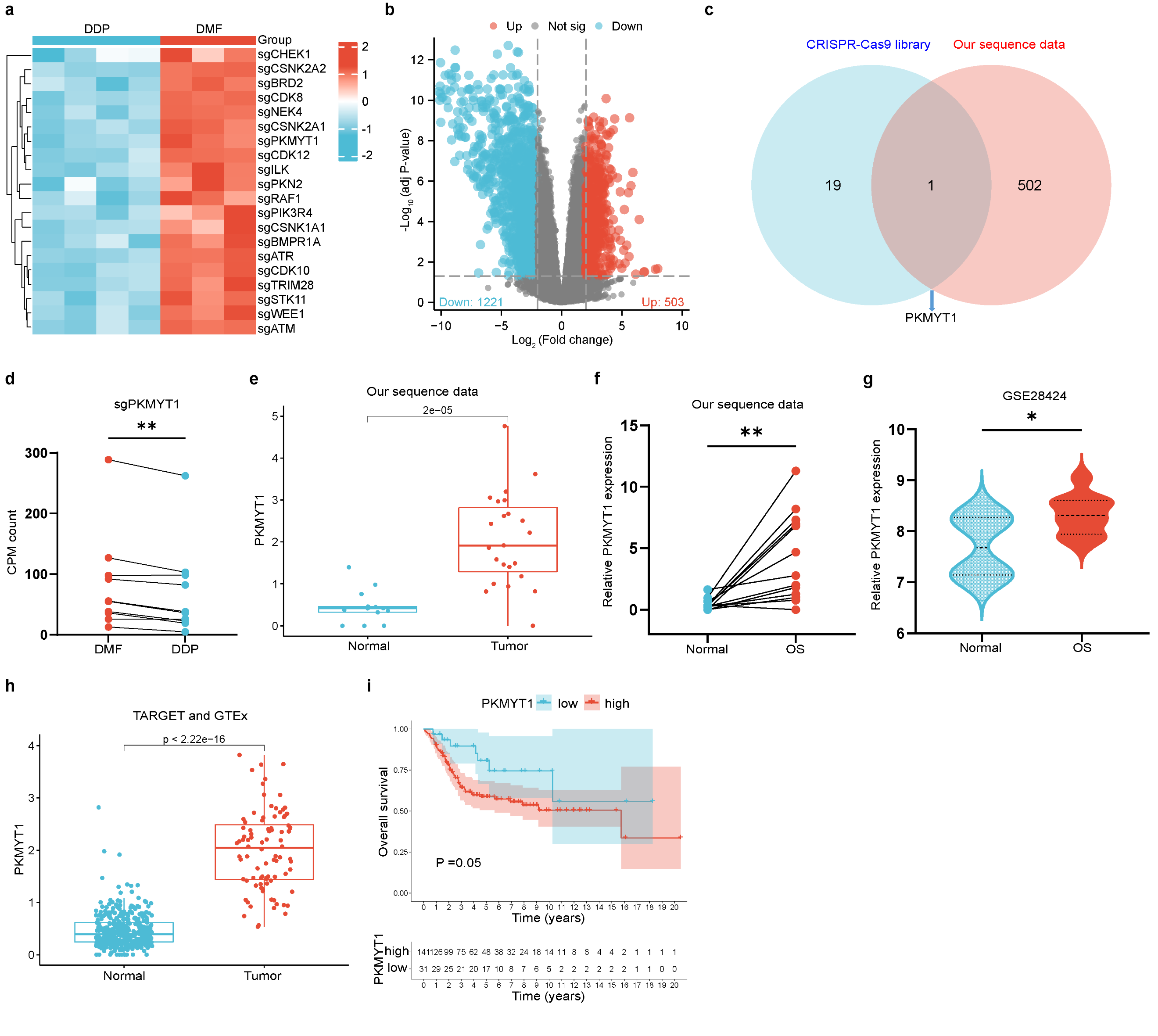


**Supplementary Fig. 1 The relationship between PKMYT1 and OS.**

a. The top 20 negatively enriched kinases identified by CRISPR-Cas9 library screen. b. Transcriptome sequencing was performed on 23 OS samples and 13 paired adjacent normal tissue samples. A volcano plot was used to depict the differential expression of genes with a p-value < 0.05 and an absolute fold change > 2. c. Venn diagram illustrating significantly upregulated genes in OS and the top 20 negatively enriched target genes. d. Comparison of CPM values of PKMYT1 sgRNA between DDP and DMF treatment groups. e. The differences in PKMYT1 expression between 23 OS samples and 13 adjacent normal tissue samples. f. Analysis of PKMYT1 expression in OS tissue and normal tissue based on TARGET and GTEx public databases. g. Analysis of PKMYT1 expression levels in OS tissue and normal tissue based on the GSE28424 public dataset. h. The differences in PKMYT1 expression between 13 pairs of OS samples and 13 adjacent normal tissue samples. i. The prognostic significance of PKMYT1 in OS was analyzed based on GSE16091 and GSE21257 cohorts from the GEO database, as well as the TARGET cohorts. Data shown are mean ± SD Error bars, *p < 0.05, **p < 0.01, ***p < 0.001, ****p < 0.0001.


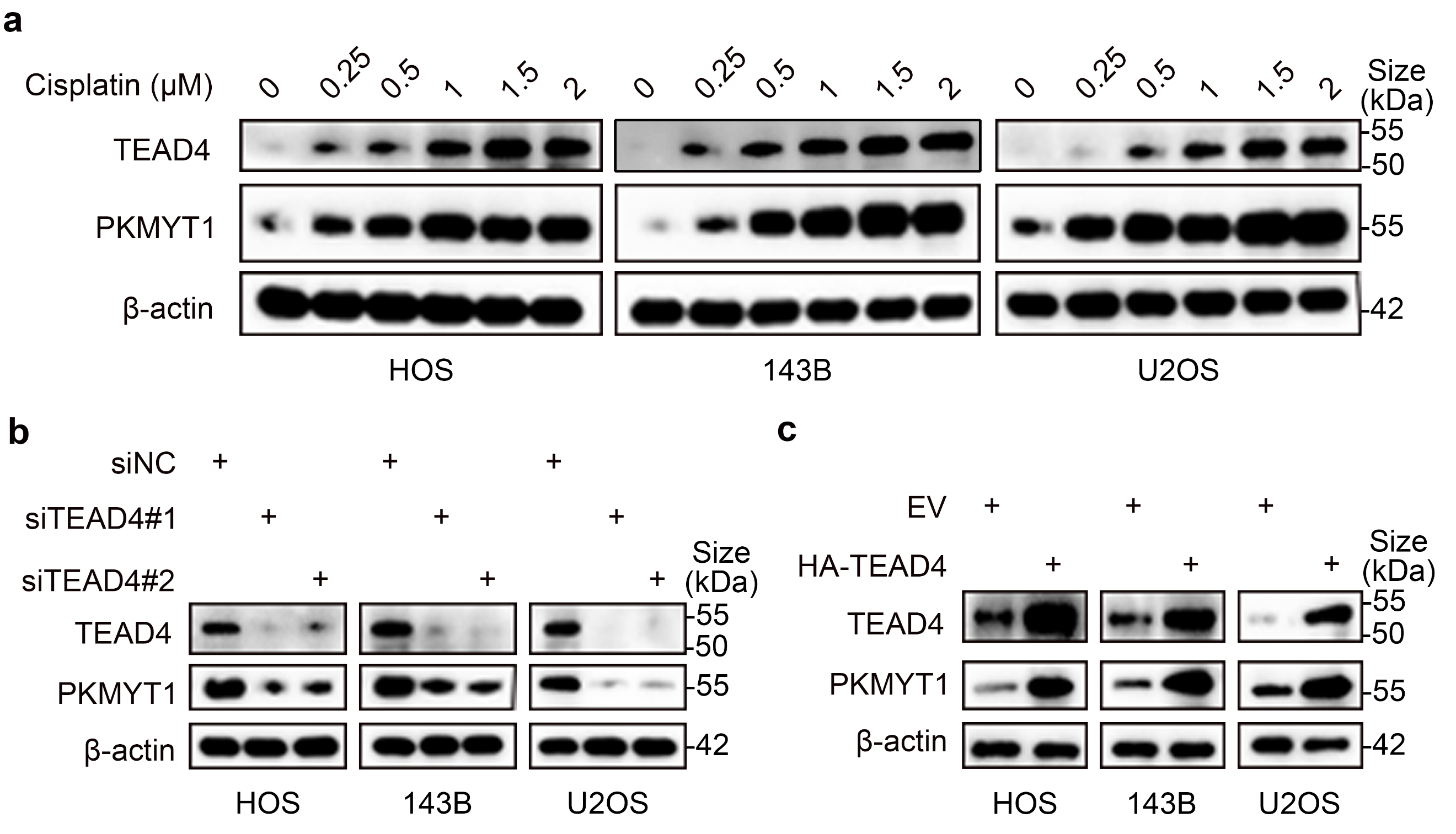


**Supplementary Fig. 2 DDP regulates PKMYT1 expression by upregulating TEAD4.**

a. HOS, 143B, and U2OS cells were treated with a series of concentrations of cisplatin for 24 h before harvesting the cells for Western blot. b. HOS, 143B, and U2OS cells were transfected with siNC, siTEAD4#1 and siTEAD4#2 for 48 h before harvesting the cells for Western blot. c. HOS, 143B, and U2OS cells were transfected with EV and HA-TEAD4 for 24 h before harvesting the cells for Western blot.


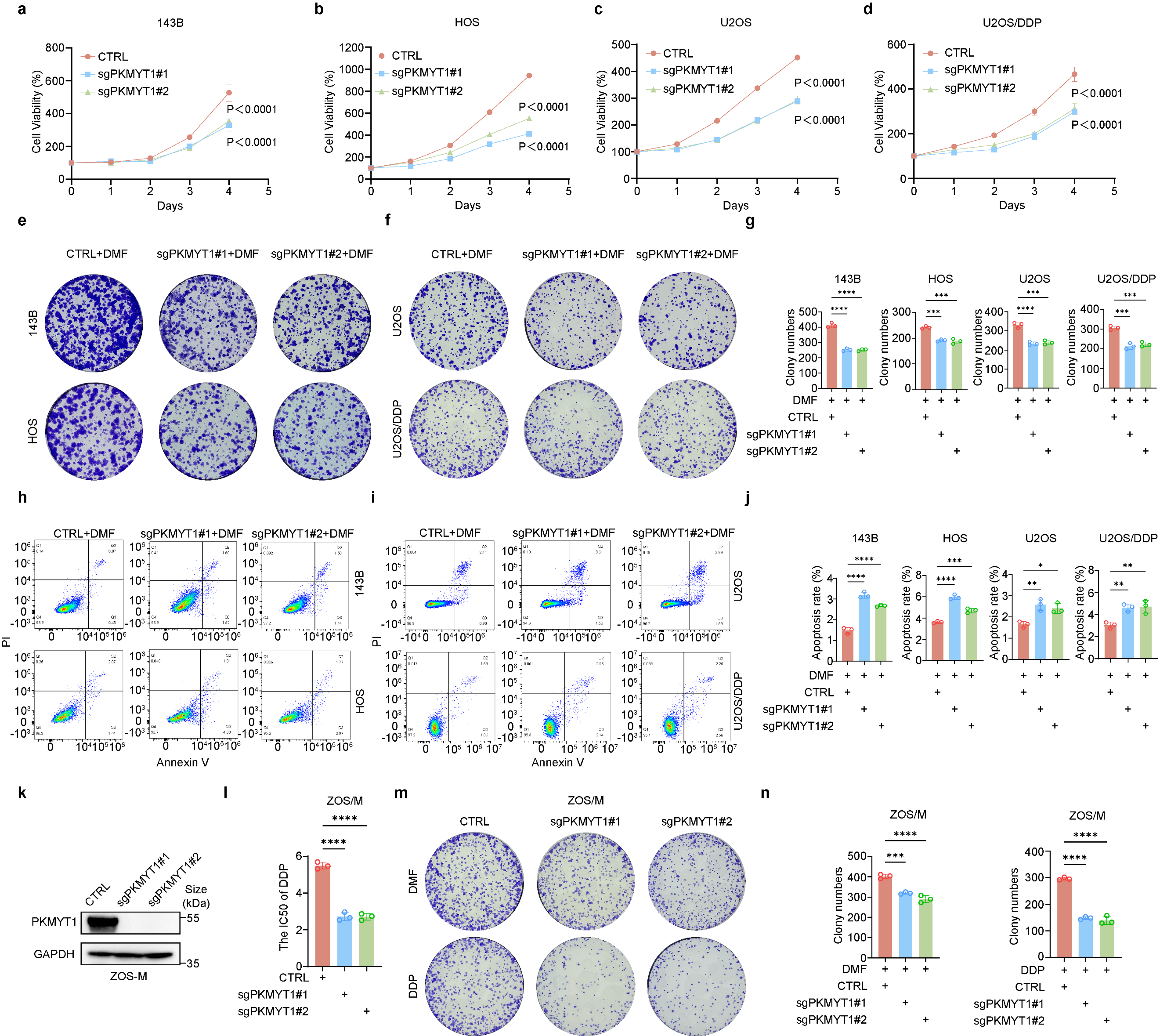


**Supplementary Fig. 3 The impact of PKMYT1 on the proliferative capacity of OS and the validation of its effect on OS to DDP sensitivity in the ZOS/M cell line.**

a-d. The proliferation capacity of OS cells after PKMYT1 knockout was measured by MTT assay (a: 143B, b: HOS, c: U2OS, and d: U2OS/DDP). e-f. Colony-forming units of PKMYT1 knockout OS cells (143B, HOS, U2OS, and U2OS/DDP) treated with vehicle measured by colony formation assay. g. Quantification of relative colony-forming units of OS cells from (e-f). h-i. The apoptosis analysis of PKMYT1 knockout OS cells (143B, HOS, U2OS, and U2OS/DDP) was treated with vehicle measured by flow cytometry assay. j. Quantification of relative apoptosis rate of OS cells from (h-i). k-n. Validating the role of PKMYT1 on OS to DDP sensitivity in the metastatic OS cell lines (ZOS/M). (k) The Efficiency of sgRNA mediated PKMYT1 knockout in ZOS/M was determined by Western blot. (l) The IC50 of ZOS/M was calculated after the addition of the gradient concentration of DDP for 24h. P values were assessed by the one-way ANOVA. (m) OS cells from d were subjected to treatment with indicated doses of vehicle for 10-14 days. Cell survival was determined by colony formation assay. (n) Quantification of relative colony-forming units of OS cells from (m). Data shown are mean ± SD Error bars, *p < 0.05, **p < 0.01, ***p < 0.001, ****p < 0.0001.


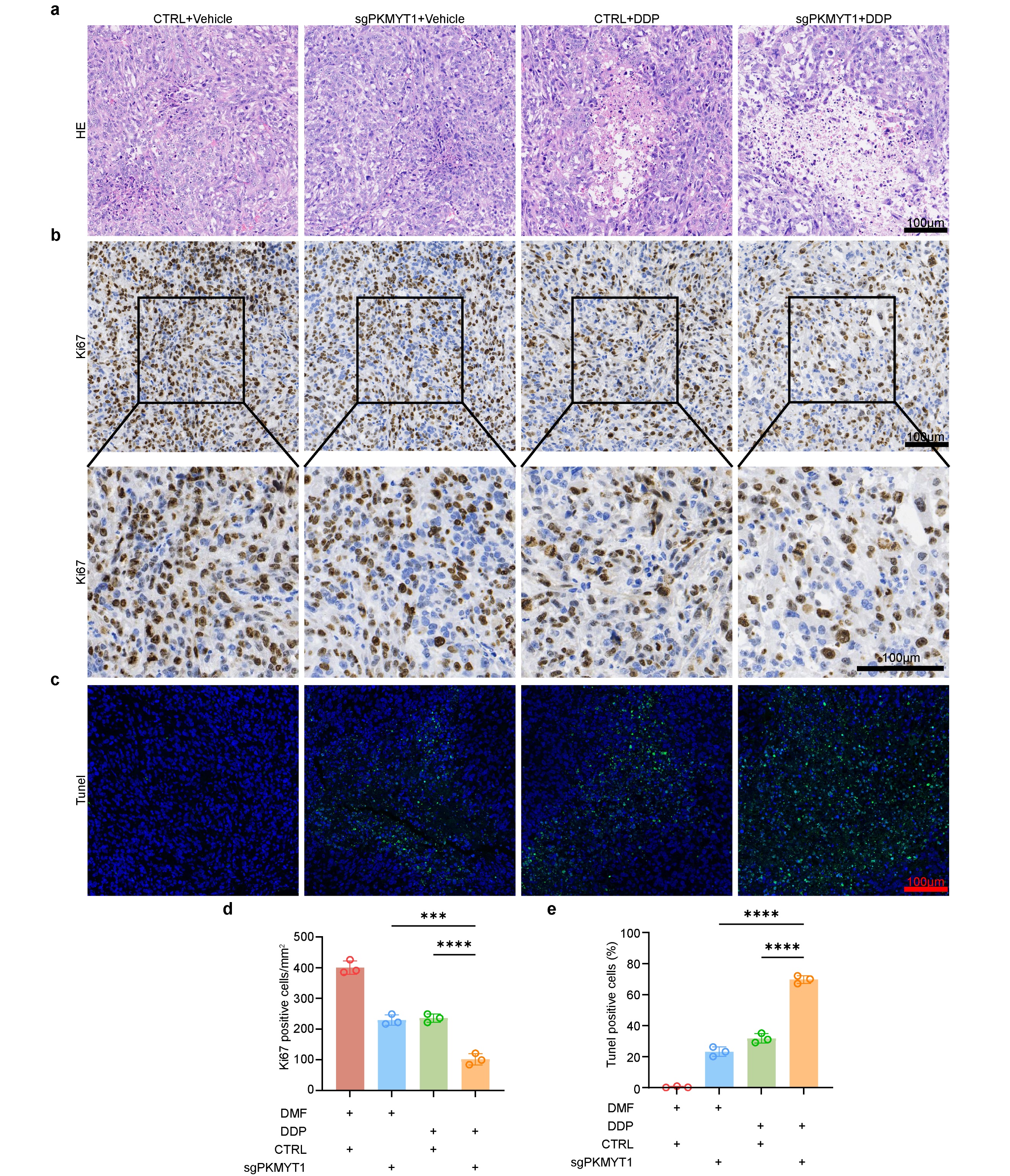


**Supplementary Fig. 4 PKMYT1 knockdown improves the sensitivity of 143B xenografts to DDP.**

a. Representative HE-stained images of tumors from Fig. 2j. b. Representative immunohistochemical images Ki67 in the indicated xenografts tumors from Fig. 2j. c. Representative immunofluorescence images of Tunnel in the indicated xenograft tumors from Fig. 2j. d. Quantitative analysis of Ki67 staining results. e. Quantitative analysis of TUNEL assay results. Data shown are mean ± SD Error bars, *p < 0.05, **p < 0.01, ***p < 0.001, ****p < 0.0001.


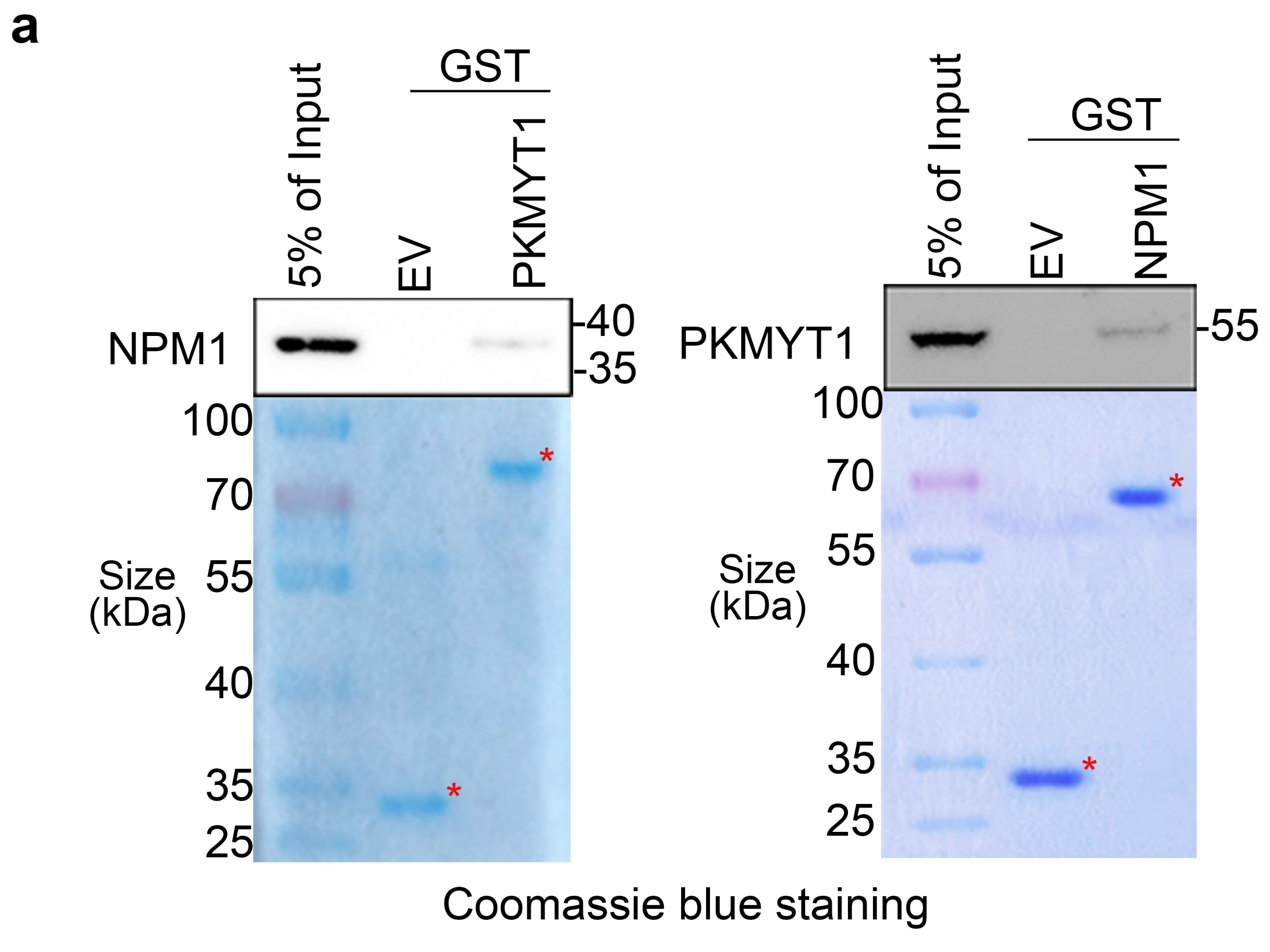


**Supplementary Fig. 5 GST-pulldown assay demonstrating the Interaction between NPM1 and PKMYT1.**

**
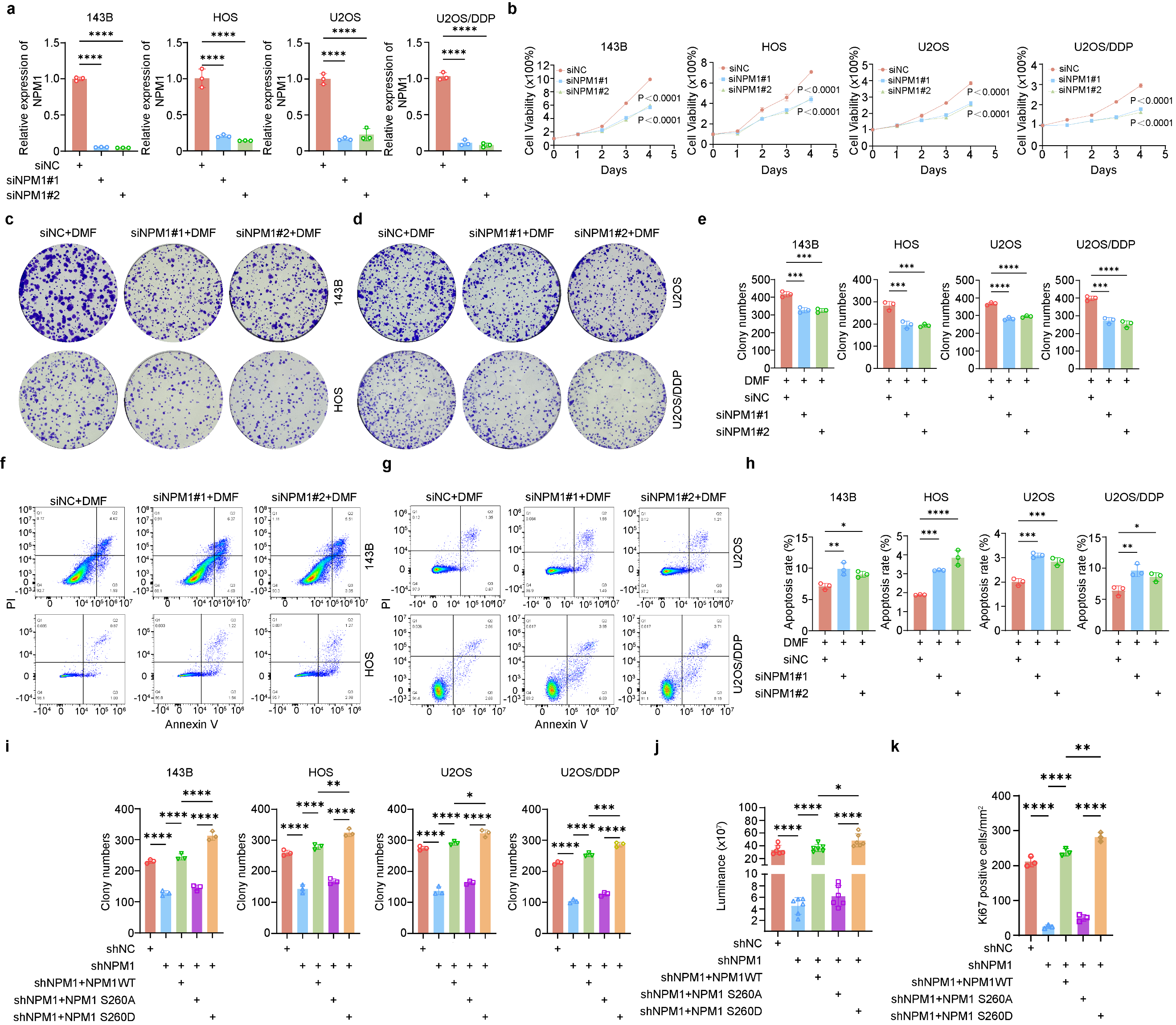
**

**Supplementary Fig. 6 The impact of NPM1 on the proliferative capacity of OS.**

a. The Efficiency of siRNA mediated NPM1 knockdown in 143B, HOS, U2OS, and U2OS/DDP was determined by RT-qPCR. b. The proliferation capacity of OS cells after NPM1 knockdown was measured by MTT assay. c-d. Colony-forming units of NPM1 knockdown OS cells (143B, HOS, U2OS, and U2OS/DDP) treated with vehicle measured by colony formation assay. e. Quantification of relative colony-forming units of OS cells from (c-d). f-g. The apoptosis analysis of NPM1 knockdown OS cells (143B, HOS, U2OS, and U2OS/DDP) was treated with vehicle measured by flow cytometry assay. h. Quantification of relative apoptosis rate of OS cells from (f-g). i. Quantification of relative colony-forming units of OS cells from Fig. 4m. j-k. Automated quantification of bioluminescence and Ki67 in the indicated xenograft tumors from Fig. 4n. Data shown are mean ± SD Error bars, *p < 0.05, **p < 0.01, ***p < 0.001, ****p < 0.0001.


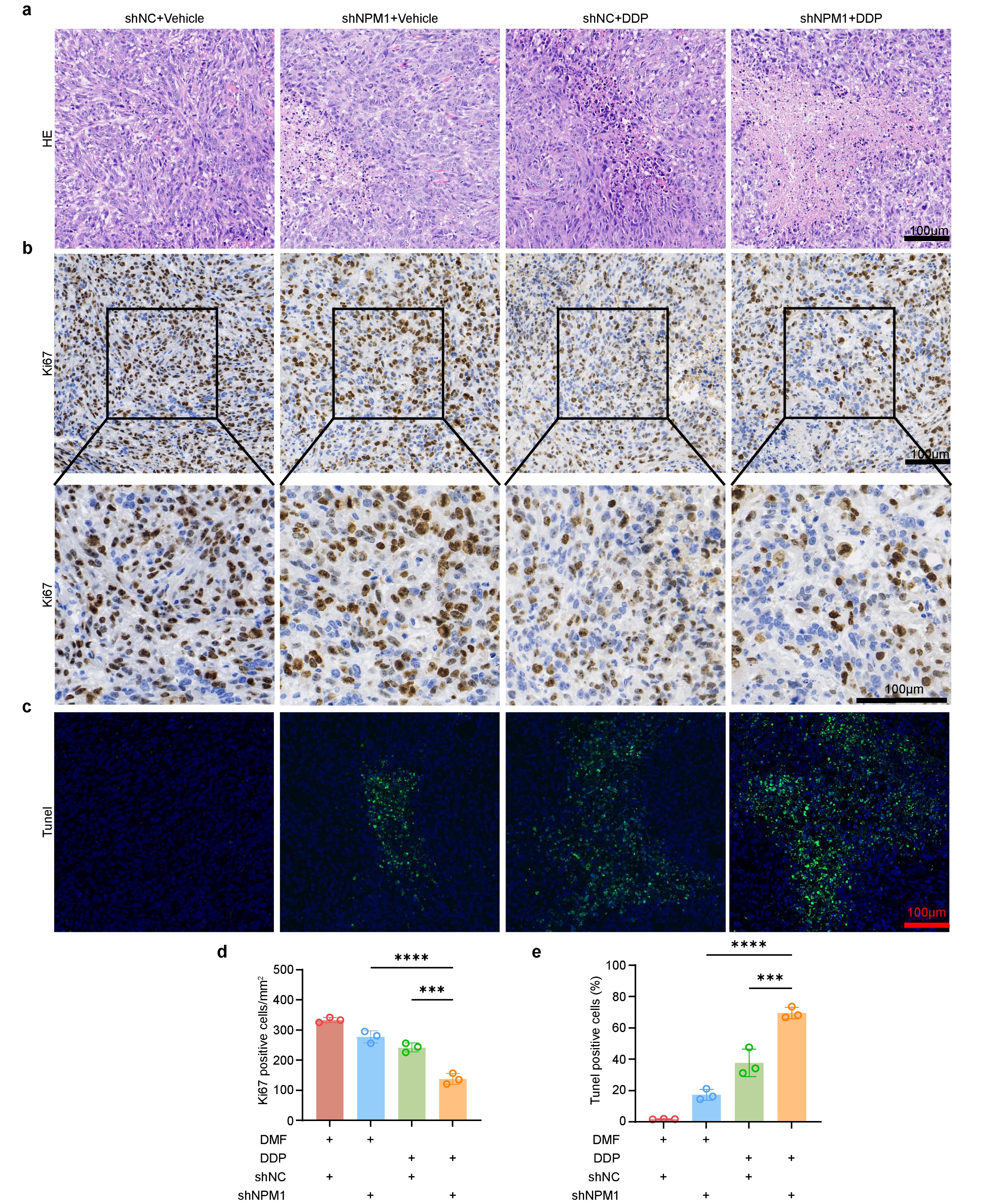


**Supplementary Fig. 7 NPM1 knockdown affects the sensitivity of 143B xenografts to DDP.**

a. Representative HE-stained images of tumors from Fig. 4I. b. Representative immunohistochemical images Ki67 in the indicated xenografts tumors from Fig. 4i. c. Representative immunofluorescence images of Tunnel in the indicated xenograft tumors from Fig. 4i. d. Quantitative analysis of Ki67 staining results. e. Quantitative analysis of TUNEL assay results. Data shown are mean ± SD Error bars, *p < 0.05, **p < 0.01, ***p < 0.001, ****p < 0.0001.


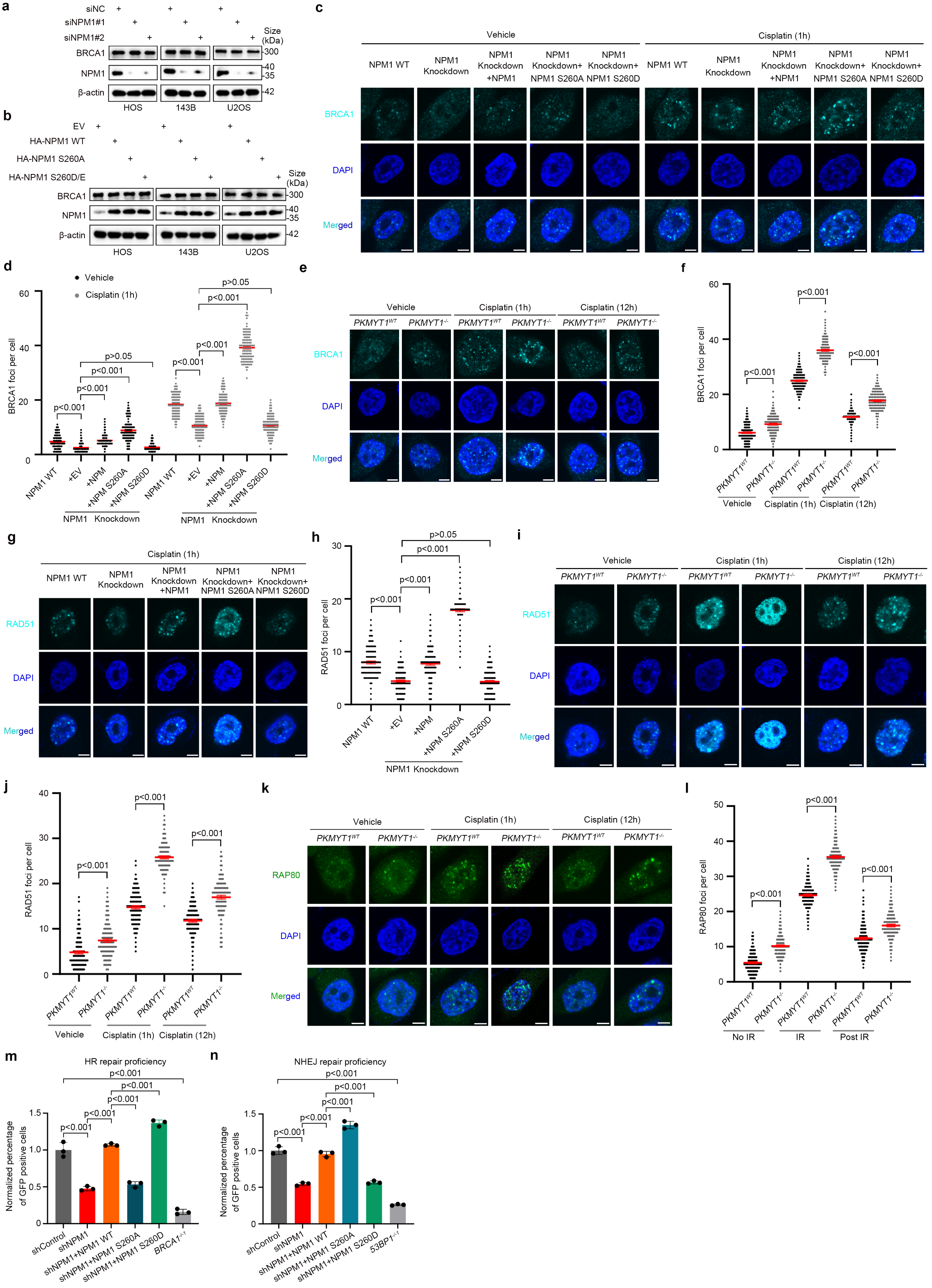


**Supplementary Fig. 8 PKMYT1-induced NPM1 S260 phosphorylation promotes efficient DSB repair.**

a. HOS, 143B, and U2OS cells transfected with siNC, siNPM1#1 and siNPM1#2 for 48 h before harvesting the cells for Western blot. b. HOS, 143B, and U2OS cells with transfected with indicated plasmids for 24 h before harvesting the cells for Western blot. c-d. Immunofluorescence analysis of the effect of NPM1 S260 phosphorylation on cisplatin-induced BRCA1 foci. NPM1 knockout U2OS cells were rescued with either wild-type NPM1, NPM1 S260A mutant and NPM1 S260D mutant, with or without cisplatin (1 μM) treatment. Representative images are shown in C, and the number of BRCA1 foci per group was calculated in d. Data are expressed as the mean ± SEM from three biological replicates. Statistical analysis was performed using Student’s t-test, p-value as indicated. Approximately 100 cells were counted per group. Scale bar, 10 μm. e-f. Immunofluorescence analysis of PKMYT1 wild-type and PKMYT1 knockout U2OS cells with or without cisplatin (1 μM) treatment. Representative immunofluorescence images were shown in e, and the number of BRCA1 foci per group was calculated in f, p-value as indicated. Scale bar, 10 μm. g-h. NPM1 knockout U2OS cells were rescued with indicated plasmids for 24 h, and immunofluorescence analysis of cells exposed to 1 μM cisplatin and recovered for 1 hour. Representative immunofluorescence images were shown in g, and the number of RAD51 foci per group was shown in h, with p-value as indicated. Scale bar, 10 μm. i-j. Immunofluorescence analysis of PKMYT1 wild-type and PKMYT1 knockout U2OS cells with or without cisplatin (1 μM) treatment. Representative immunofluorescence images were shown in i, and the number of RAD51 foci per group was calculated in j. Scale bar, 10 μm. k-l. Immunofluorescence analysis of PKMYT1 wild-type and PKMYT1 knockout U2OS cells with or without cisplatin (1 μM) treatment. Representative immunofluorescence images were shown in k, and the number of RAP80 foci per group was calculated in l, p-value as indicated. Scale bar, 10 μm. m. NPM1 Knockout HEK293T cells were rescued by NPM1 WT, NPM1 S260A or NPM1 S260D, and then cells were subjected to HR assay, with BRCA1 knockout as control. Data are presented as the mean ± SEM from three replicates. Each group included over 1000 cell counts. P-value as indicated. n. NPM1 Knockout HEK293T cells were rescued by NPM1 WT, NPM1 S260A or NPM1 S260D, and then cells were subjected to NEHJ assay, with BRCA1 knockout as control. Data are presented as the mean ± SEM from three replicates. Each group included over 1000 cell counts, p-value as indicated.


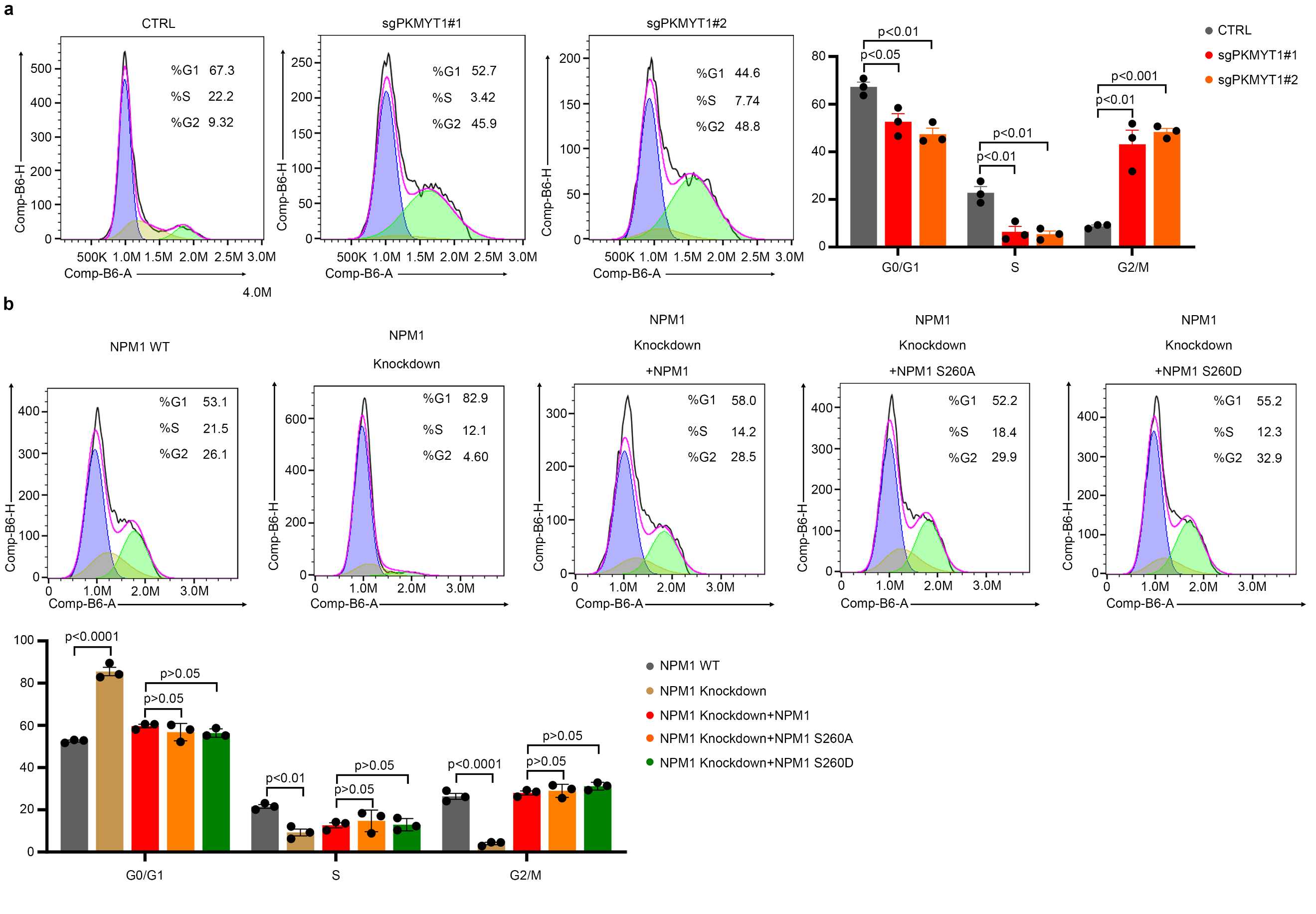


**Supplementary Fig. 9 The effect of PKMYT1 knockout, NPM1 knockdown, and NPM1 S260 phosphorylation statute on the cell cycle of OS cells.**

a. The impact of PKMYT1 knockout on the cell cycle of OS cells. b. NPM1 Knockout OS cells were rescued by NPM1 WT, NPM1 S260A or NPM1 S260D, and then cells were subjected to cell cycle assay.


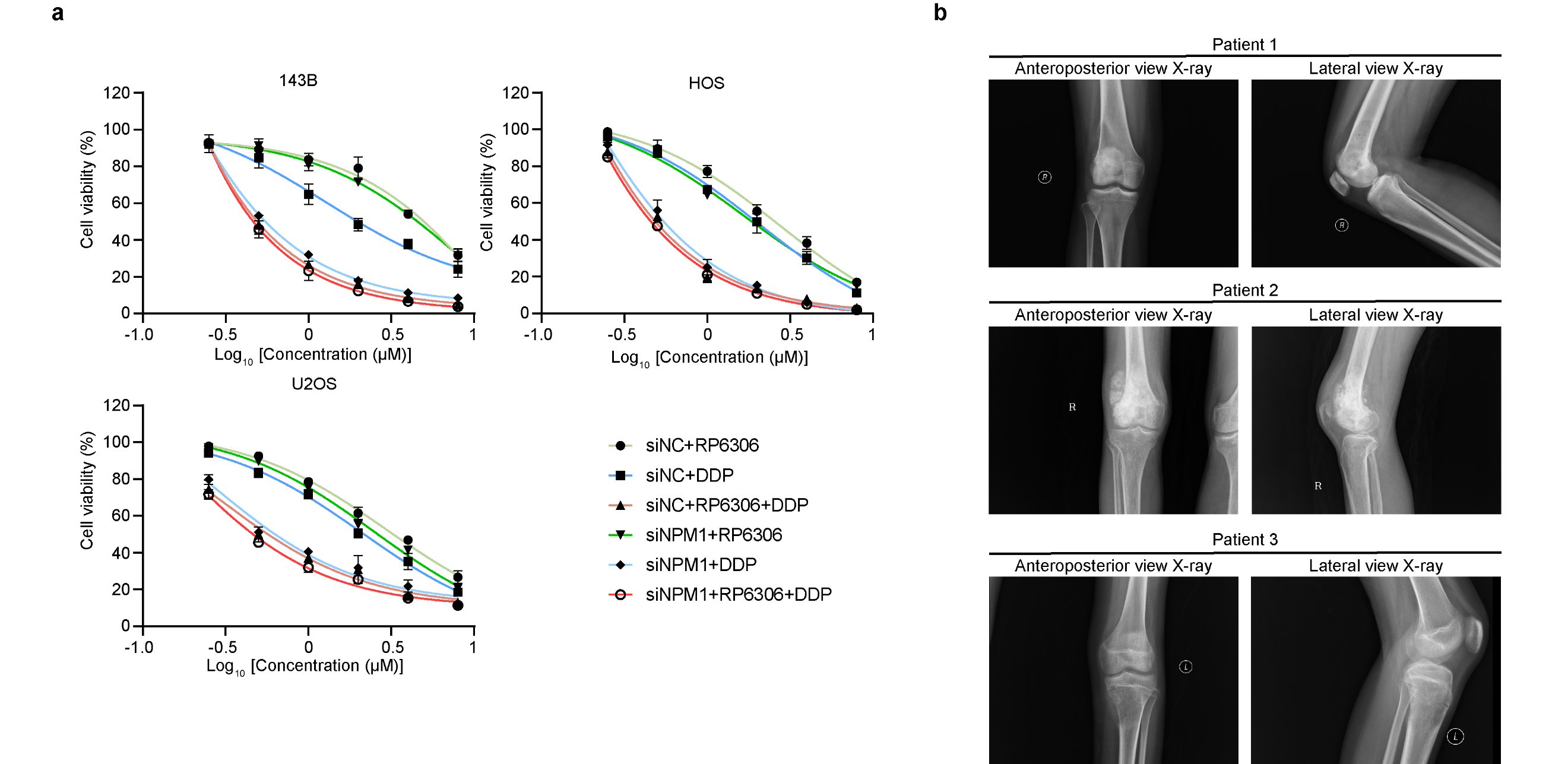


**Supplementary Fig. 10 Effect of NPM1 knockdown on the combined treatment of RP6306 and DDP and the clinical information of OS patients used for constructing organoids.**

a. The effect of NPM1 knockdown on the combined treatment of RP6306 and DDP. b. The X-ray photo of OS patients used for constructing organoids.
