# Supplementary material for: PKMYT1 kinase ameliorates cisplatin sensitivity in osteosarcoma
Source: Signal Transduct Target Ther. 2025 May 21;10:165. doi: 10.1038/s41392-025-02250-7 (PMC12092789; doi:10.1038/s41392-025-02250-7)

Figure 1M

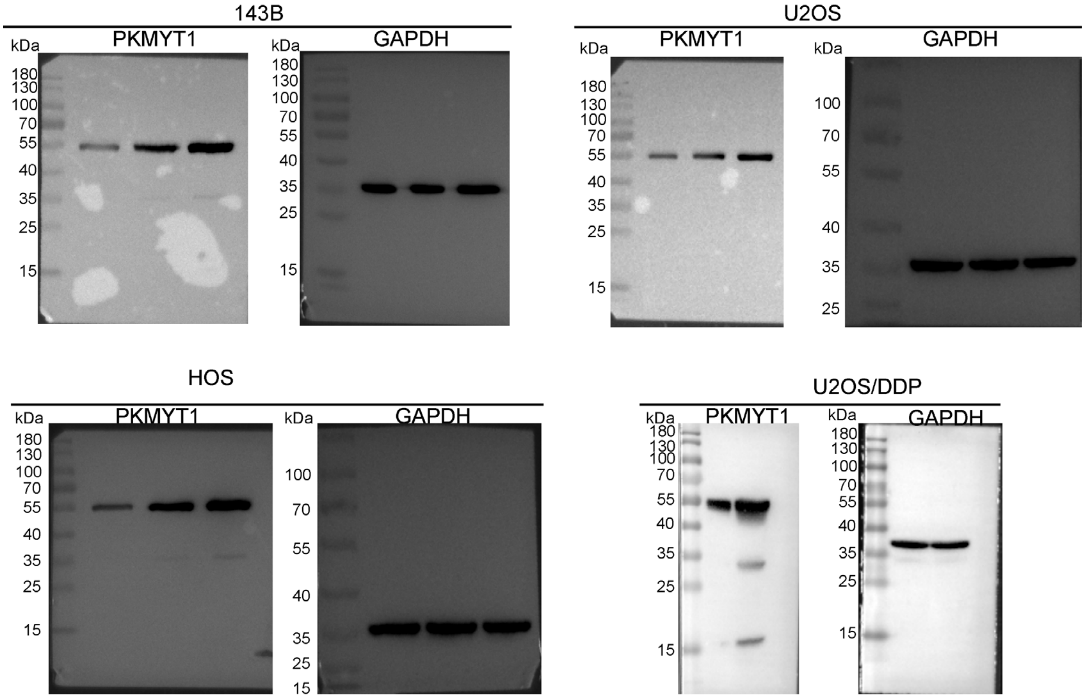

143B
PKMYT1
GAPDH
U2OS
PKMYT1
GAPDH
HOS
PKMYT1
GAPDH
U2OS/DDP
PKMYT1
GAPDH
kDa
180
130
100
70
55
40
35
25
15

Figure 2A

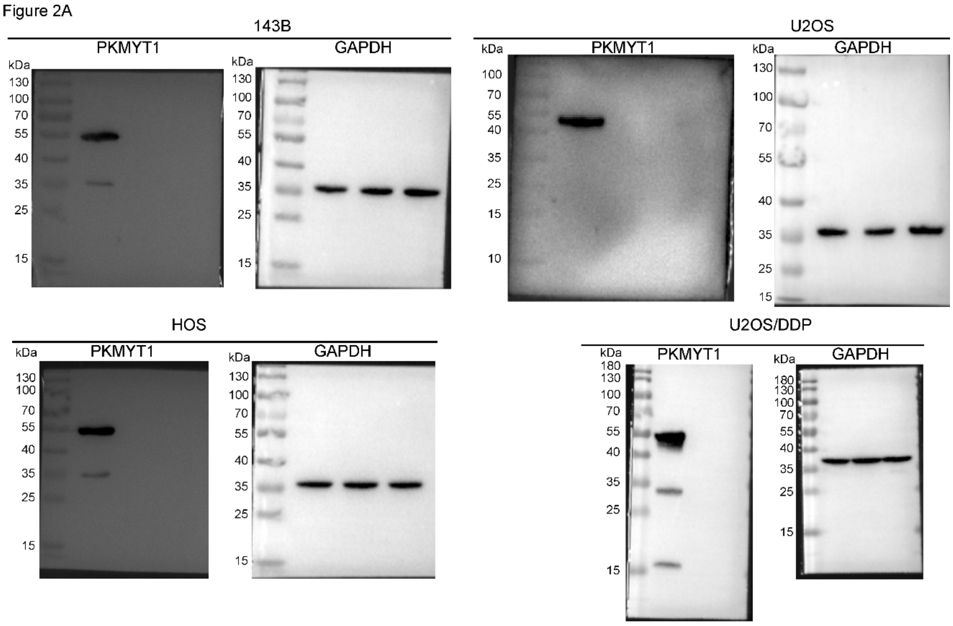

143B
PKMYT1
GAPDH
U2OS
PKMYT1
GAPDH
HOS
PKMYT1
GAPDH
U2OS/DDP
PKMYT1
GAPDH
kDa

Figure 3

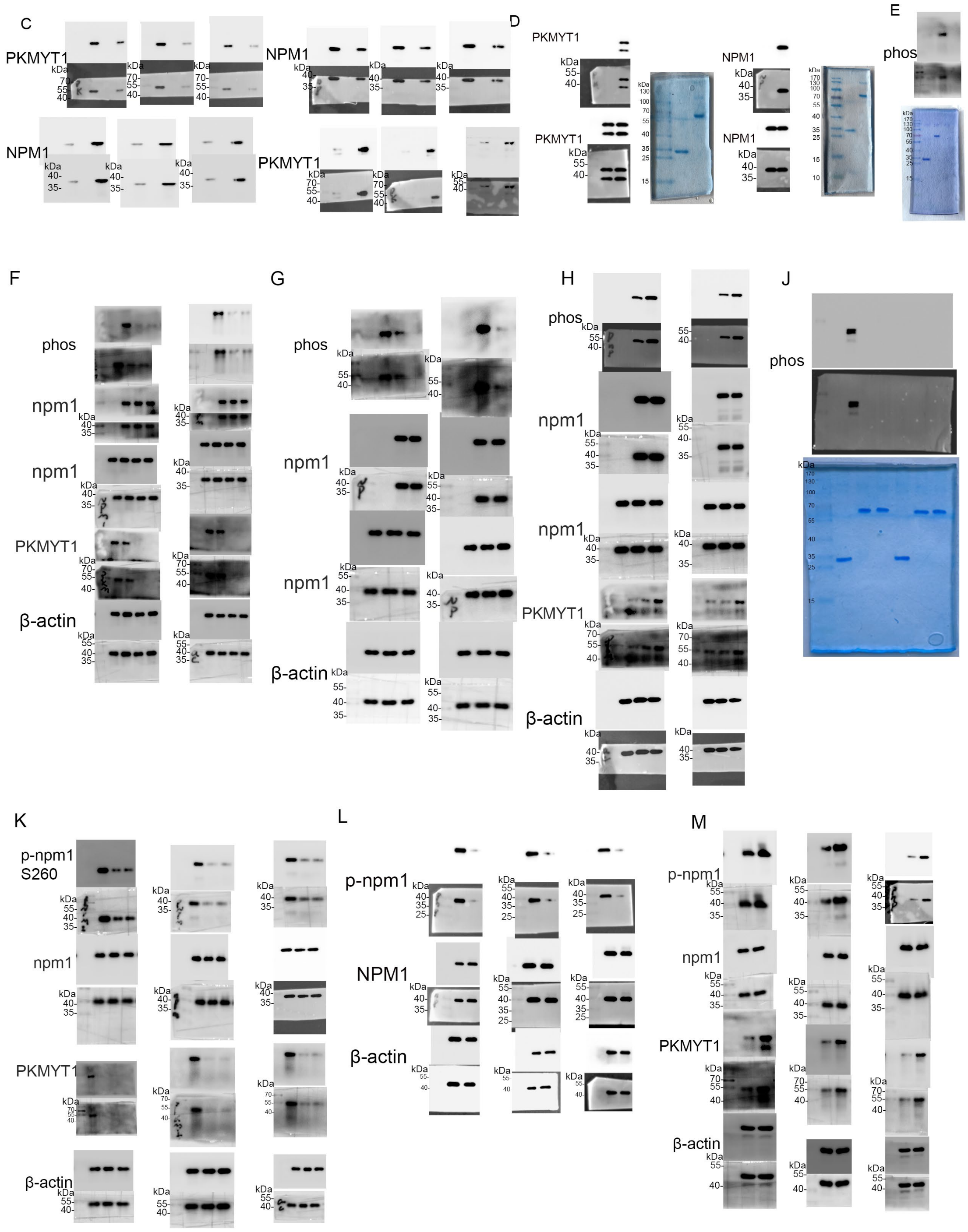

C
PKMYT1
NPM1
D
PKMYT1
NPM1
E
phos
F
phos
npm1
npm1
PKMYT1
β-actin
G
phos
npm1
npm1
β-actin
H
phos
npm1
npm1
PKMYT1
β-actin
J
phos
K
p-npm1 S260
npm1
PKMYT1
β-actin
L
p-npm1
NPM1
β-actin
M
p-npm1
npm1
PKMYT1
β-actin

Figure 4A

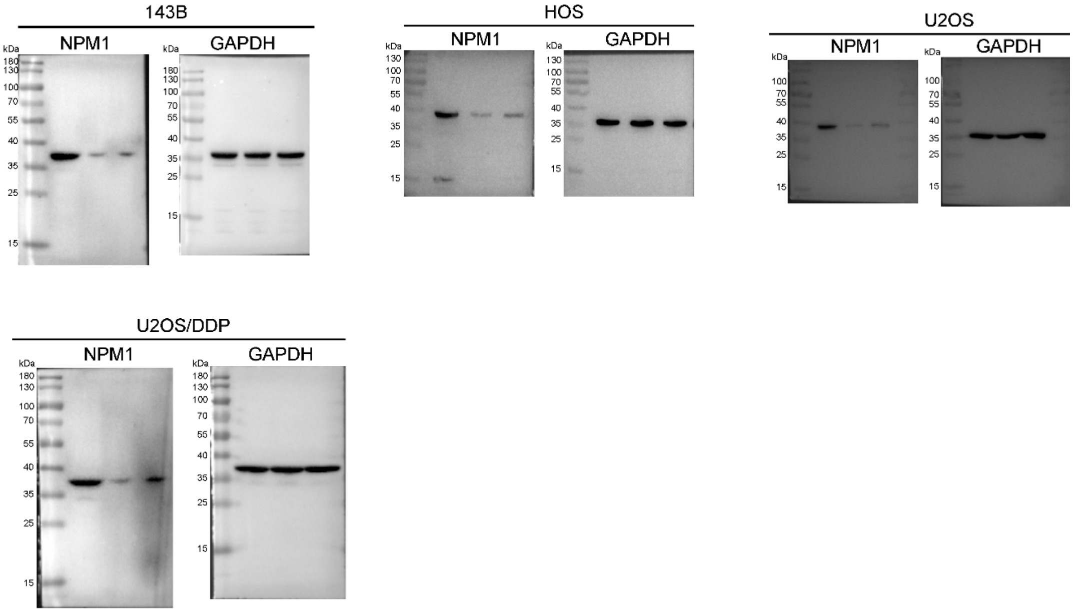
143B
NPM1
GAPDH
HOS
NPM1
GAPDH
U2OS
NPM1
GAPDH
U2OS/DDP
NPM1
GAPDH
kDa

Figure 5

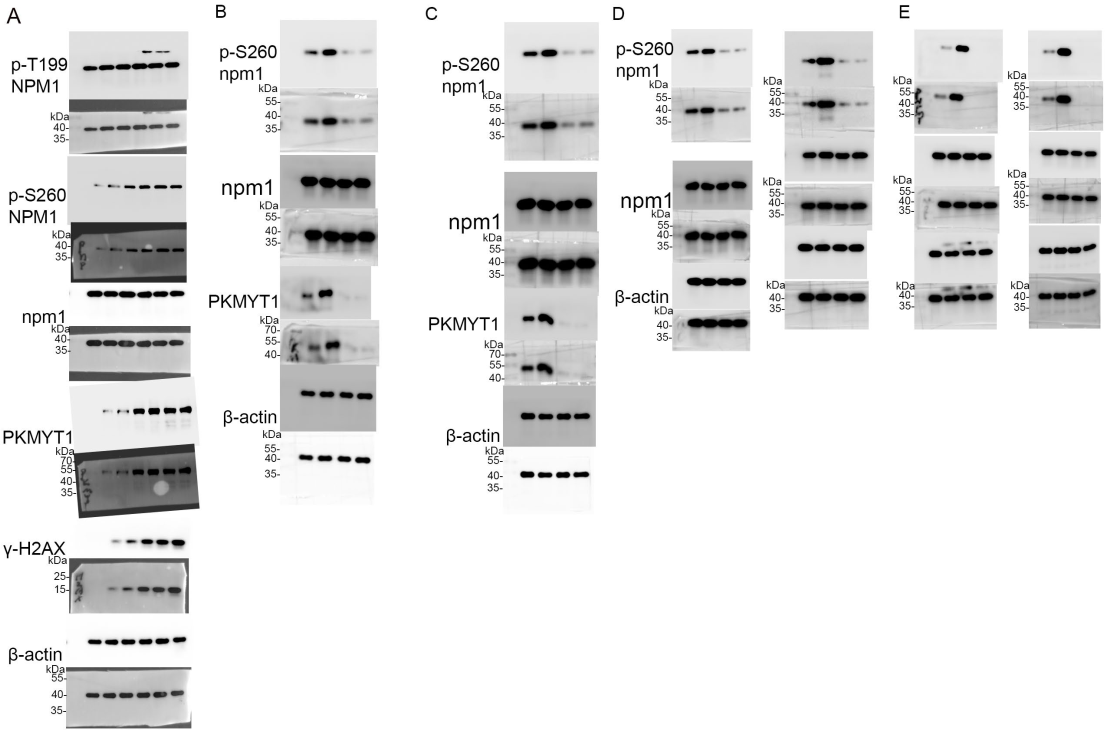

A
p-T199
NPM1
p-S260
NPM1
npm1
PKMYT1
γ-H2AX
β-actin
B
p-S260
npm1
npm1
PKMYT1
β-actin
C
p-S260
npm1
npm1
PKMYT1
β-actin
D
p-S260
npm1
npm1
β-actin
E

Figure 5

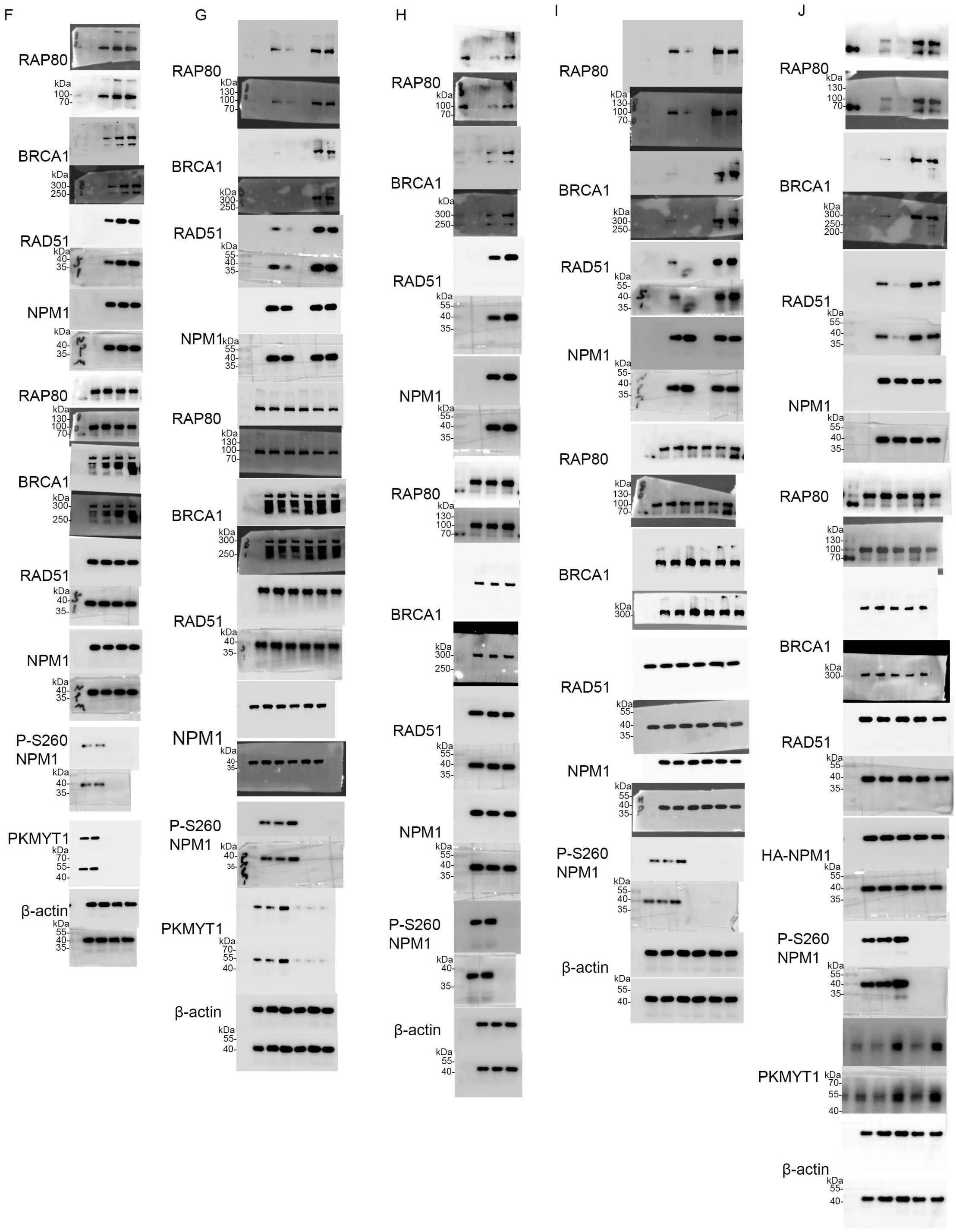
F
RAP80
BRCA1
RAD51
NPM1
RAP80
BRCA1
RAD51
NPM1
P-S260 NPM1
PKMYT1
β-actin
G
RAP80
BRCA1
RAD51
NPM1
RAP80
BRCA1
RAD51
NPM1
P-S260 NPM1
PKMYT1
β-actin
H
RAP80
BRCA1
RAD51
NPM1
RAP80
BRCA1
RAD51
NPM1
P-S260 NPM1
β-actin
I
RAP80
BRCA1
RAD51
NPM1
RAP80
BRCA1
RAD51
NPM1
P-S260 NPM1
β-actin
J
RAP80
BRCA1
RAD51
NPM1
RAP80
BRCA1
RAD51
HA-NPM1
P-S260 NPM1
PKMYT1
β-actin
kDa

Figure 6

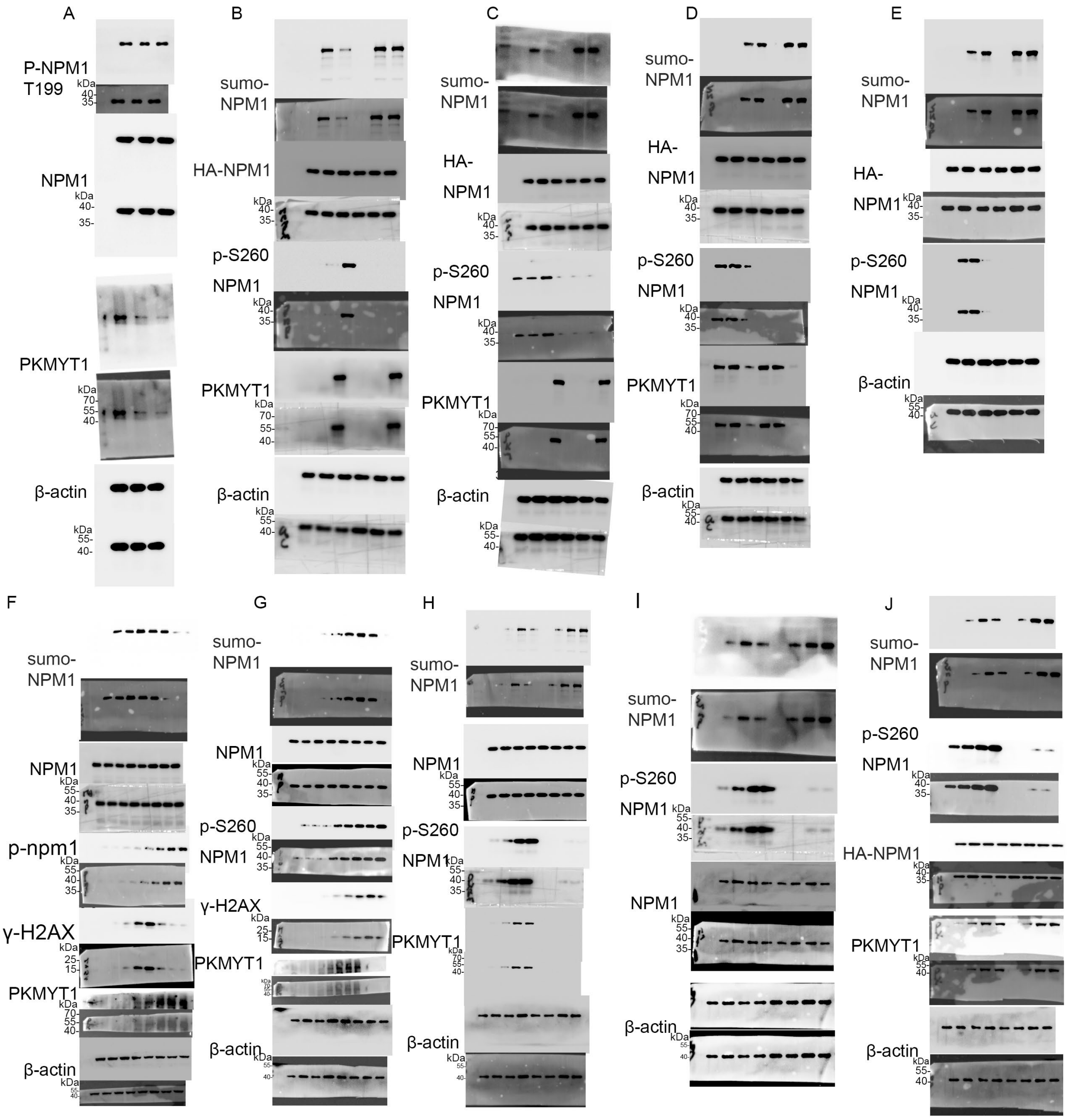
A
P-NPM1
T199
NPM1
PKMYT1
β-actin
B
sumo-
NPM1
HA-NPM1
p-S260
NPM1
PKMYT1
β-actin
C
sumo-
NPM1
HA-
NPM1
p-S260
NPM1
PKMYT1
β-actin
D
sumo-
NPM1
HA-
NPM1
p-S260
NPM1
PKMYT1
β-actin
E
sumo-
NPM1
HA-
NPM1
p-S260
NPM1
β-actin
F
sumo-
NPM1
NPM1
p-npm1
γ-H2AX
PKMYT1
β-actin
G
sumo-
NPM1
NPM1
p-S260
NPM1
γ-H2AX
PKMYT1
β-actin
H
sumo-
NPM1
NPM1
p-S260
NPM1
PKMYT1
β-actin
I
sumo-
NPM1
p-S260
NPM1
NPM1
β-actin
J
sumo-
NPM1
p-S260
NPM1
HA-NPM1
PKMYT1
β-actin

Supplementary Figure 2

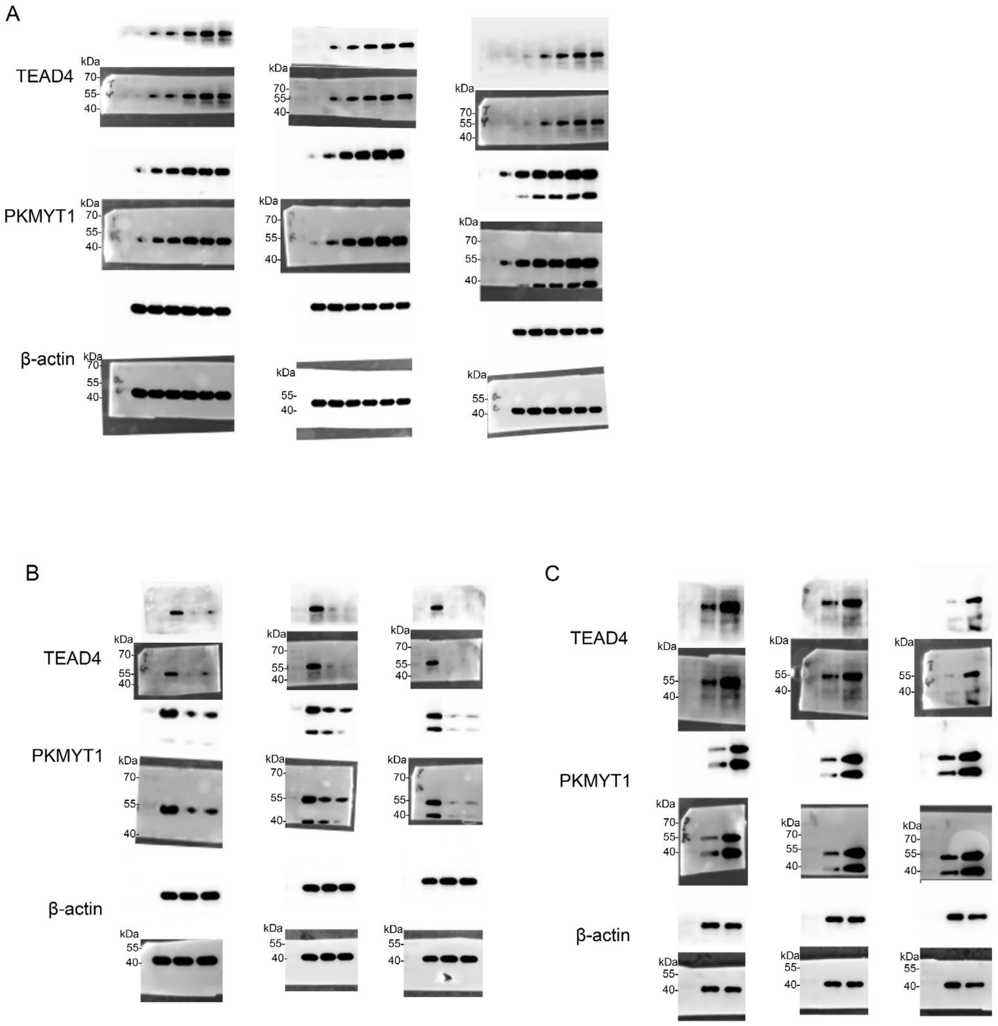

Supplementary Figure 3

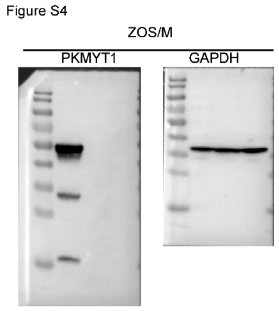

Supplementary Figure 5

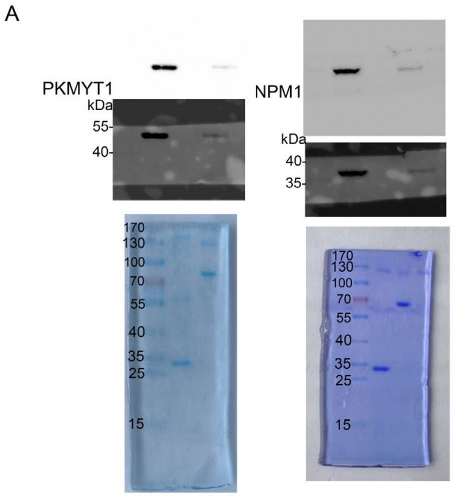

Supplementary Figure 8

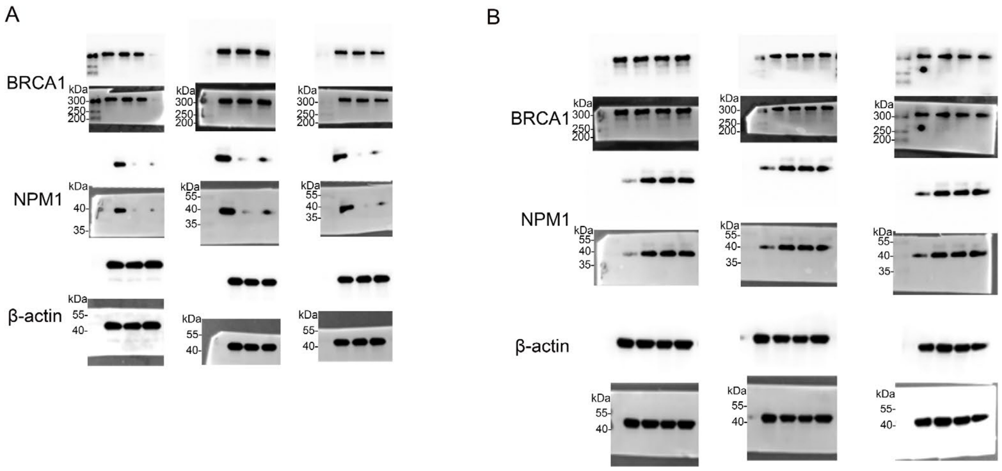

Supplement: Supplementary file 2 — Original western blots [file 41392_2025_2250_MOESM2_ESM.pdf]
